# Mind the gap! Addressing unresolved aspects of abuse potential evaluation and scheduling of classic and novel psychedelic drugs

**DOI:** 10.1177/02698811251382147

**Published:** 2025-10-16

**Authors:** David J. Heal, Jane Gosden, Sharon L. Smith

**Affiliations:** 1 Department of Life Sciences, University of Bath, UK; 2 DevelRx Ltd, BioCity, Nottingham, UK

**Keywords:** psychedelics, abuse potential, controlled drug scheduling, Controlled Substances Act, safety pharmacology

## Abstract

Psychedelic research is progressing at breakneck speed and is creating new challenges for drug developers, regulatory authorities, and legislators. Most “classic” psychedelics undergoing clinical investigation are C-I controlled drugs with perceived high potential for abuse and no medical use. These and next-generation psychedelic drug-candidates require scientific and clinical assessment of their abuse and dependence potential before transitioning into a controlled drug schedule assigned to clinically approved drugs (C-II to C-V). Food and Drug Administration is likely to undertake the first regulatory assessment of a “classic” psychedelic, and it has led in disseminating advice on how to address the clinical and regulatory challenges. We have built on this foundation by discussing areas of abuse and dependence evaluation procedures that remain unclear or have not previously been covered. Psychedelic drug-candidates can be classified into three categories, that is, “classic” (well-known compounds including psilocybin, *N,N*-dimethyltryptamine and lysergic acid diethylamide) and “novel” psychedelics (e.g., analogues of known psychedelics), and located between them is what we describe as “grey area” psychedelics (e.g., non-hallucinogenic 5-HT_2A_ agonists). In this review, we set out clear proposals for categorizing psychedelic drug-candidates, describe the development pathway and abuse/dependence testing procedures appropriate to each, and, finally, offer our perspective on how these drugs will be evaluated and scheduled under the auspices of the U.S. Controlled Substances Act. Although we used the United States as a test case, the principles and analyses we used and the screening framework for assessing the abuse potential of psychedelic drug-candidates are universally applicable and can be easily adapted to the regulatory requirements and procedures in other countries.

## Introduction

Initial commercial interest in developing psychedelics as medicinal products focused on patentable variants of known compounds, for example, psilocybin (e.g., Compass Pathways, Cybin, and Usona Institute), lysergic acid diethylamide (LSD) (MindMed and MindBio Therapeutics), *N,N*-dimethyltryptamine (DMT) (Small Pharma/Cybin), and 5-methoxy-N,N-dimethyltryptamine (5MeO-DMT) (GH Research, Beckley Psytech, and BioMind Labs). More recent developments include the development of novel analogues of known psychedelics (e.g., MindSet/Otsuka Pharmaceutical, Cybin, Gilgamesh Pharmaceuricals), and non-hallucinogenic inducers of neuroplasticity (e.g., IntraCellular Therapies/Johnson & Johnson, Gilgamesh Pharmaceuticals, and BetterLife Pharma). Hence, there are psychedelic drugs undergoing clinical evaluation, or on the brink of entering clinical development, which are well-known C-I psychedelic compounds, novel 5-HT_2A_ receptor agonists that have psychoactive properties with varying degrees of similarity to known C-1 psychedelics, and totally novel molecules. The latter include the group of the non-hallucinogenic 5-HT_2A_ receptor agonists, which are predicted to lack the abuse risks posed by the hallucinogenic C-I psychedelics, but have not themselves been studied in sufficient depth to define their individual risks for abuse and/or dependence.

An evaluation of abuse potential is a key part of the non-clinical and clinical safety assessment that has to be performed on drugs that act on the central nervous system (CNS) that are seeking approval for medical use in humans. In the United States, guidance for the pharmaceutical industry has been published by the Center for Drug Evaluation and Research (CDER) of the Food and Drug Administration (FDA) (CDER/FDA, 2017). Although the CDER/FDA (2017) guidance is comprehensive and informative, the pharmacological and psychoactive properties of the psychedelics pose serious challenges for some tests, for example, placebo-blinding for abuse potential trials in drug-experienced human volunteers, and pose technical challenges for the conduct of others, for example, intravenous self-administration studies in animals. To address these challenges, scientists from the Controlled Substance Staff Division (CSS) of FDA have published several articles describing their evolving view on how to assess the abuse potential of psychedelic compounds for medical use (Bonson, 2018; Calderon et al., 2018, 2023). In 2023, CDER/FDA issued a discussion document setting out the points that pharmaceutical companies should consider when developing psychedelic drug-candidates (CDER/FDA, 2023)

The CDER/FDA (2023) guidance applies to the development of psychedelics and entactogens, for example, 3,4-methylenedioxymethamphetamine. It defines “classic” psychedelics with the examples of psilocybin and LSD. CDER/FDA (2023) states that these drugs are “5-HT_2_ receptor agonists.” Although the hallucinogenic effects of the psychedelics derive from 5-HT_2A_ receptor agonism, there are safety implications surrounding the interaction of these compounds with 5-HT_2B_ and 5-HT_2C_ receptor subtypes (see Heal et al., 2023).

The CDER/FDA (2023) guidance states that for certain “classic” psychedelics, a wealth of knowledge exists based on published research and real-world experience that is sufficient for CSS/FDA to make its determination of the abuse potential, safety risks and level of Controlled Substances Act (CSA) scheduling without the need to conduct specialized non-clinical and clinical studies to revisit the topic. Based on the examples cited in the CDER/FDA (2023) guideline, psilocybin and LSD fall into this category.

Where this level of non-clinical and clinical evidence is lacking, the CDER/FDA (2023) guidance advises drug developers to conduct a full abuse potential assessment, as described by CDER/FDA (2017).

With the exception of post-marketing data to define the real-world level of the abuse of a drug, the human abuse potential (HAP) trial is considered by CDER/FDA to provide the most sensitive and accurate test to predict the risk of human abuse after the drug has been approved. CDER/FDA (2023) states that an HAP study may not be required for “*certain psychedelic drugs*” when “*the subjective effects predictive of abuse are well characterized from extensive clinical studies and robust epidemiological data exist to demonstrate that individuals are using the psychedelic drug for abuse purposes*.” Conducting an HAP trial on a psychedelic drug-candidate poses substantial challenges, as described by Calderon et al. (2023). First, it is challenging to include a viable placebo control arm in the trial because the psychoactive effects of hallucinogenic psychedelics are so profound. Second is the thorny issue of selecting an appropriate active comparator (approved medical use and in C-II to C-V) to serve positive control for the study. Since no psychedelic drug has yet been approved for medical use, the selection would have to be based on the similarity of psychoactive effects, which would limit the selection to phencyclidine (PCP) (C-II) or ketamine (C-III). Despite the fact that the subjective experiences induced by N-methyl-D-aspartate (NMDA) antagonists and 5-HT_2A_ receptor agonists are quite different (Gouzoulis-Mayfrank et al., 2005), both types of agents induce profound sensory, perceptual, and time distortions psychotic-like effects, so it may be feasible to adopt this approach for some psychedelics. However, it will not be appropriate when it comes to evaluating the abuse potential of the next generation of drugs, which includes the non-hallucinogenic 5-HT_2A_ receptor agonist drug-candidates.

From this short summation, a picture emerges that the psychedelics have injected a sense of purpose into CNS drug research that has remained in the doldrums since the heyday of the introduction of the Selective Serotonin Reuptake Inhibitors (SSRIs) and atypical antipsychotics. While there can be no question that the psychedelics offer enormous benefits in a wide range of therapeutic indications, their unique pharmacological properties create challenges when designing experimental techniques, risk profile criteria, and regulatory procedures to ensure that if, and when, psychedelic compounds gain approval for medical use, they are assigned controlled drug scheduling that is fair and appropriate to the risk that they pose to patients and the general public.

It is also clear that while the regulatory agencies have started to disseminate guidance on how to set about evaluating the abuse and dependence potential of the “classic” psychedelics, for example, psilocybin/psilocin, LSD, and DMT, the pathway for evaluating the next generation of psychedelic drug-candidates is far less well defined.

In previous reviews, we have offered our views on how to discover and develop this next generation of psychedelic drug-candidates (Heal et al., 2023), how to adapt the non-clinical testing to evaluate their potential for abuse and dependence (Heal et al., 2018), and the regulatory pathway for assessing the abuse risks and scheduling of the current generation of psychedelics (Henningfield et al., 2022, 2023). In this review, we have looked at the areas of abuse potential assessment that remain to be elucidated, how innovations in psychedelic research will pose new challenges, how they could be addressed, and offer suggestions for controlled drug scheduling as the current crop of psychedelics enters the market, and we enter the next phase which will be the development of the next generation of psychedelic drug-candidates.

## U.S. Controlled Substances Act (CSA)

We chose to focus on the development and regulatory approval of psychedelics as medicines in the United States because it is the country where most pharmaceutical companies will be seeking the first approval of their drug-candidates. In addition, the FDA has published comprehensive guidelines on the evaluation of drug abuse and dependence, and it has clearly defined process for determination of controlled drug classification (CDER/FDA, 2017), and it has published advice on how to address the clinical and regulatory challenges posed by the psychedelics (Calderon et al., 2018, 2023; CDER/FDA, 2023).

In contrast, European Medicines Agency (EMA) published its guidance on assessing the abuse risks in 2006 (in Europe they are described as “dependence” risks; CHMP/EMA, 2006), but have not updated them to adapt to the development of the psychedelics. There are substantial differences between the European and United States approaches to abuse potential evaluation and controlled drug decision-making (Calderon et al., 2015), for example, European regulators do not rely on findings from HAP trials (though they will of course consider the findings if an HAP study has been performed to support a U.S. drug registration) and controlled drug status and scheduling are devolved to individual countries. Nevertheless, the European nations are signed up to the 1971 United Nations Convention on Psychotropic Substances Treaty that was designed to control the possession, manufacture, and distribution of psychoactive drugs including psychedelics. The classic and novel synthetic psychedelics are classified as controlled drugs with no medical use in Europe, and therefore, the legislative and bureaucratic impediments to psychedelic research and their development as medicines will be similar to those of the United States.

The U.S. CSA classifies known psychoactive drugs according to their established risk for abuse and the personal and public harms that could derive from it. In the case of novel CNS-active substances, this classification is based on an estimate of the human abuse risk together with an assessment of the adverse outcomes that could arise from such abuse (https://www.dea.gov/drug-information/csa). There are five schedules within the CSA: Schedules 2 to 5 (C-II to C-V) are applied to drugs with abuse potential that have been approved for medical or veterinary use. These schedules apply restrictions on the manufacture, distribution (including import and export), and prescribing of controlled drugs with C-II being the highest level of restriction and C-V the lowest. Schedule 1 (C-I) is limited to a drug or other substance that “(*i) has a high potential for abuse, (ii) has no currently accepted medical use in treatment in the United States and (iii) there is a lack of accepted safety for use of the drug or other substance under medical supervision*.” (https://uscode.house.gov/view.xhtml?req=38&f=treesort&num=5465). The use of the phrase “*high potential for abuse*” is often mistakenly assumed to imply that drugs and substances in C-I pose a greater risk for abuse, dependence and safety than drugs in C-II to C-V, whereas in reality, C-I is a catch-all for drugs spanning a wide spectrum of risks and harms. Thus, within C-I are highly dangerous substances, for example, heroin and various fentanyl analogues, drugs with moderate harms, for example, cannabis, and even substances with negligible risks. As an example, cannabidiol (CBD), which is not psychoactive and has no abuse potential, is classified as a C-I substance when extracted from cannabis plants. Thus, Epidiolex™, which is CBD from the *Cannabis sativa* plant, had to be temporarily placed in C-V when it was approved to treat epilepsy before it was subsequently de-scheduled in April 2020.

A special licence issued by the Drug Enforcement Administration is required to possess, produce, or supply any substance in C-I, and the bureaucratic hurdles involved in obtaining a C-I licence constitute a significant barrier to conducting non-clinical or clinical research.

## The classification of psychedelics

Psychedelics produce a temporary altered state of consciousness that is characterized by a range of effects including sensory, perceptual, and temporal distortions, a dissociative state, loss of self-identity, sensations of boundlessness, and oneness with the world (Johnson et al., 2008; Lerner and Lyvers, 2006; Mortaheb et al., 2024). These effects are dependent upon the type of psychedelic and dose used. The prominent visual distortions produced by psychedelics often consist of flowing geometric visual patterns (Bressloff et al., 2002) that can evolve into complex hallucinations involving objects, animals, people, or landscapes. Subjects often interpret the psychedelic experience as a mystical event with transformative and healing properties (Lerner and Lyvers, 2006).

Psychedelics come from three major chemical classes, that is, the tryptamines, ergolines, and phenethylamines. However, given the intense interest in the medical benefits offered by the psychedelics, it is highly likely that some novel chemical scaffolds will be employed in the search for more pharmacologically selective and patentable psychedelic drug-candidates.

Most well-known psychedelics are naturally occurring molecules produced by plants, for example, DMT and mescaline, fungi, for example, psilocybin and LSD, or animals, for example, bufotenine, and occasionally by species from multiple kingdoms, for example, 5MeO-DMT, which is present in various plants and animals. A wide range of psychedelic 5-HT_2A_ receptor agonists have been synthesized including the widely used tool compounds, for example, 2,5-dimethoxy-4-iodoamphetamine (DOI) and 2,5-dimethoxy-4-methylamphetamine (DOM).

Although most known psychedelics functionally interact with a multiplicity of CNS receptors (Ermakova et al., 2022; Holze et al., 2024; Rickli et al., 2016; Warren et al., 2024), it is widely believed that activation of the 5-HT_2A_ receptor subtype produces the characteristic cluster of psychoactive effects that comprise the psychedelic experience. Early indirect evidence pointing to 5-HT_2_ receptors as the pharmacological mediator came from the observation of an excellent correlation between the affinity of various indoleamines and phenethylamines and their potency to induce hallucinations in humans (Glennon et al. 1984; Titeler et al., 1988). Later experiments conducted with the 5-HT_2A_ receptor antagonist, ketanserin, established that the 5-HT_2A_ subtype was responsible for the psychedelic, subjective, and neural effects of LSD in human subjects (Holze et al., 2020; Preller et al., 2017, 2018, 2019).

**Figure 1.**
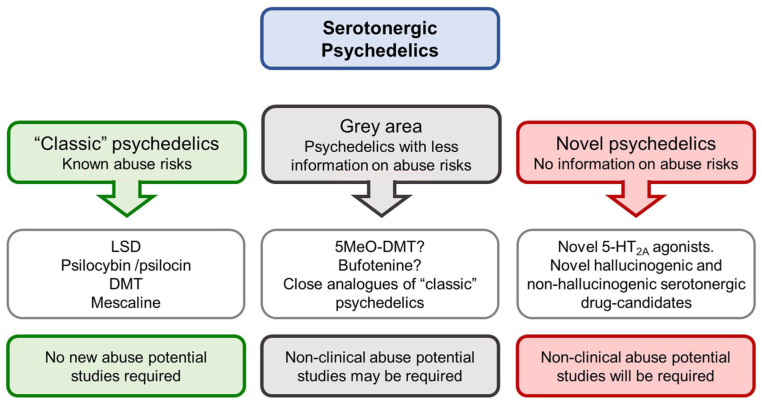
Classification of psychedelic drug-candidates. We have sub-divided the serotonergic psychedelics into three categories: classic, novel, and grey area. According to the CDER/FDA (2023) draft guidance, the classic psychedelics are compounds where a wealth of knowledge exists to define their abuse potential and safety risks. Novel psychedelic compounds that pose unknown risks for abuse potential and safety will require a full non-clinical assessment, as described by CDER/FDA (2017). The grey area psychedelics are those with less comprehensive data on abuse potential and safety, or novel molecules which close analogues of the classic psychedelics. For these drug-candidates, the route for abuse potential assessment is unclear. CDER: Center for Drug Evaluation and Research; FDA: Food and Drug Administration.

The current landscape on psychedelic drug research and development together with regulatory advice on the abuse potential evaluation for CSA scheduling (CDER/FDA, 2023) supports the proposal that psychedelic drug-candidates can be classified under three broad categories, as shown in Figure 1.

**Figure 2.**
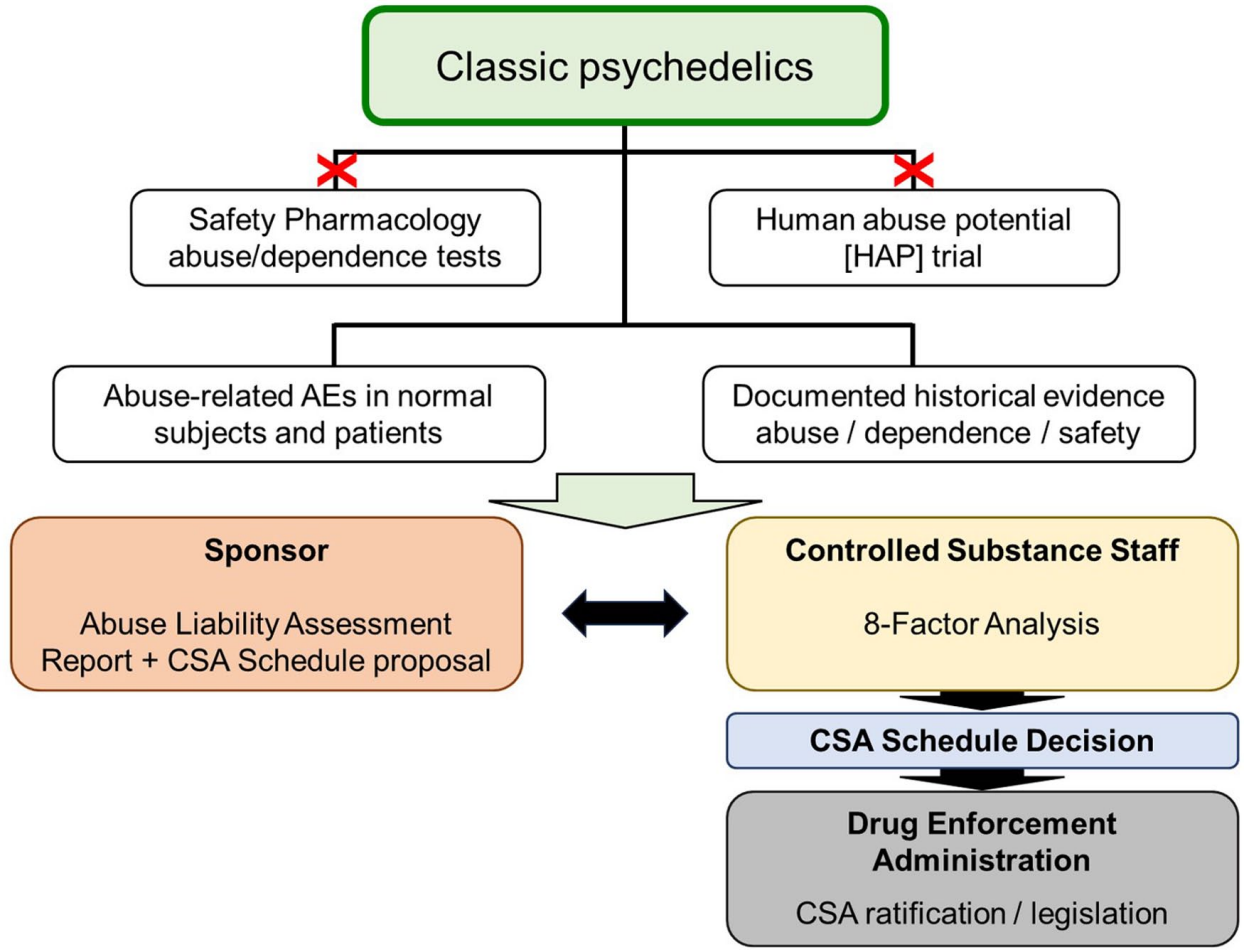
Proposed regulatory framework for the abuse potential evaluation and CSA scheduling of classic psychedelic drugs in the United States. This schematic shows the proposed path for assessing the abuse potential and CSA scheduling for the classic psychedelics. The CDER/FDA (2023) guidance states a wealth of knowledge and real-world experience are available to enable CSS/FDA to make its determination on CSA scheduling without the need to conduct specialized non-clinical and clinical studies to revisit the topic. CDER: Center for Drug Evaluation and Research; CSA: Controlled Substances Act; CSS: Controlled Substance Staff Division; FDA: Food and Drug Administration; X: assessments are not required.

In the United States, the development pathway for the “classic” psychedelics has been described in the CDER/FDA (2023) draft guidance, which specifically names psilocybin and LSD as examples of “classic” psychedelics. In this guidance, FDA’s stated view is the abuse potential, and safety risks posed by the “classic” psychedelics are well understood based on several thousand publications, drug abuse monitoring, and experience of their psychological, behavioural, and physiological effects gained from traditional medicine and extensive human experimentation. Given this wealth of knowledge, FDA concluded that it already had access to sufficient evidence to perform its abuse potential assessment and make a determination of the appropriate CSA schedule(s) for these compounds. Conducting additional non-clinical abuse testing and/or evaluating these specific psychedelics in HAP trials would not be informative because they are unlikely to generate meaningful new insights that will influence or contribute to the scheduling determination. Although FDA only named LSD and psilocybin/psilocin, given the large body of knowledge about DMT and mescaline, it is highly likely that these compounds will also be included in the “classic” psychedelics group. The framework for abuse potential evaluation of the “classic” psychedelics and CSA scheduling in the FDA regulatory approval process is illustrated in Figure 2.

The “novel” psychedelics category comprise drug-candidates which comply with the description in the CDER/FDA (2023) draft guidance,

For those psychedelic drugs that have not been well-characterized previously in preclinical and clinical studies, sponsors should conduct a full abuse potential assessment, as described in the guidance for industry Assessment of Abuse Potential of Drugs (CDER/FDA, 2017), before submission of a new drug application.

In short, the FDA will treat drug-candidates that are “novel” psychedelics as possessing the same uncharacterized abuse and dependence risks as any other CNS drug-candidate. This category will include drug-candidates with novel chemical structures, and/or those which differ substantially from the “classic” psychedelics in terms of their serotonergic and extended pharmacology. This group will include a wide array of pharmacological and psychoactive profiles and already includes drug-candidates that produce the typical spectrum of psychedelic effects in humans, and possibly, drug-candidates that combine the 5-HT_2A_ receptor agonist pharmacology of the conventional psychedelics with the monoamine-releasing activity of the entactogens. In addition, some companies are actively exploring a new generation of “non-hallucinogenic” drug-candidates that may deliver therapeutic efficacy without exposure to the psychedelic experience produced by conventional agents like psilocybin/psilocin, DMT, and LSD. The pharmacology of these drug-candidates will need to be thoroughly elucidated, including off-target pharmacological interactions that might support abuse or produce dependence, and they will also require investigation in the standard non-clinical safety pharmacology package of abuse tests, that is, drug discrimination and intravenous self-administration.

The CDER/FDA (2023) draft guidance states,

A human abuse potential study should generally be conducted when a drug has shown abuse-related signals in animal and/or human studies. However, a human abuse potential study may not be scientifically necessary for certain psychedelic drugs to support the abuse potential assessment in a new drug application when the subjective effects predictive of abuse are well characterized from extensive clinical studies and robust epidemiological data exist to demonstrate that individuals are using the psychedelic drug for abuse purposes.

The challenges in selecting a pharmacologically-relevant positive control and maintaining placebo-blinding when conducting HAP trials on conventional psychedelic drug-candidates have been acknowledged (Calderon et al., 2023), and the CDER/FDA (2023) draft guidance suggests an alternative approach to access evidence in humans pertaining to the abuse potential of psychedelic drug-candidates. However, the technical hurdles to conducting HAP studies will not apply to “non-hallucinogenic” drug-candidates. As stated in the CDER/FDA (2017) guidelines “*Data from HAP studies are important in developing abuse-related drug product labeling and in determining whether the drug product will be scheduled under the CSA*.” Findings from HAP trials are regarded by FDA as providing the most predictive information about the abuse risks posed by novel CNS drugs because the results have been generated by the subset of the population who are most likely to experiment with and abuse prescription drugs after they have entered the market. Therefore, if abuse-related signals are observed in animal or human studies during the development of “non-hallucinogenic” psychedelic drug-candidate, an evaluation in an HAP trial, which is the conventional route for CNS drug-candidates, would be a logical next step in the abuse assessment of this class of compound.

Located between the “classic” psychedelics and “novel” psychedelics, there exists an area of uncertainty which we have called the “grey area.” From an abuse potential evaluation perspective, drug-candidates in this category will either follow the same route as the “classic” psychedelics or they will be placed in the “novel” psychedelics category where they will be required to undergo the same program of non-clinical and clinical testing as every other novel CNS drug-candidate.

According to our assessment, the “grey area” psychedelics comprises three distinct subtypes of drug-candidate compounded with one regulatory factor:

1. Known biological and synthetic psychedelic compounds that possess psychoactive properties that differ substantially from the “classic” psychedelics.
2. Known biological and synthetic psychedelic compounds where the tolerability and safety risks in humans are inadequately established (e.g., not used in traditional medicine or religious practices, and a paucity of surveillance data on abuse and safety).
3. Novel analogues of “classic” psychedelics where a decision on the requirement for information from specialized non-clinical abuse testing and/or an HAP trial will be dependent on whether the safety, tolerability, and abuse risks are sufficiently similar to those of “classic” psychedelics to be regarded as being equivalent.
4. Divergence between the FDA and other regulatory authorities on the scientific and clinical evidence requiring an abuse potential evaluation. As an example, there are substantial differences between the United States and Europe over the data package required for an abuse potential determination, and also the procedures applied to controlled drug decision-making and scheduling (Calderon et al., 2015).

“Novel” psychedelics, which are close congeners of “classic” compounds like psilocybin/psilocin, DMT, or LSD, would logically fall into the “grey area” category, but which of the known psychedelics would CSS/FDA and other regulators consider to be potential candidates for this category? We selected 5MeO-DMT as an example of a well-known psychedelic that could be a candidate for the “grey area” classification. 5MeO-DMT is unusual because it is a psychedelic of animal and botanical origin. 5MeO-DMT is present at high concentration in the parotid and tibial glands of the Sonoran desert toad (*bufo alvarius*), where it is synthesized by the enzymatic conversion of bufotenine (5OH-DMT; Weil and Davis, 1994). Several pharmaceutical companies are investigating the therapeutic potential of 5MeO-DMT, and it is in clinical trials as a treatment for a range of psychiatric disorders (Bistue Millón et al., 2025). One of the attractions of 5MeO-DMT is its very short biological half-life, which could potentially obviate the need to administer it to patients in a lengthy session under constant supervision of two psychotherapists in a specialist clinic. The pharmacological properties of 5MeO-DMT and the psychedelic experience it evokes are very different from psilocybin/psilocin, DMT, LSD, and other “classic” compounds (Reckweg et al., 2022), which has led to 5MeO-DMT being described as an “atypical” psychedelic. The primary pharmacology of 5MeO-DMT is as an equipotent, full agonist of both the 5-HT_2A_ (EC_50_ = 26 nM) and 5-HT_1A_ (EC_50_ = 25 nM) receptors (Warren et al., 2024), which is different from the profile of the “classic” psychedelics. Like other biological psychedelics, 5MeO-DMT also incurs a raft of other receptor interactions (Holze et al., 2024; Warren et al., 2024). Users of 5MeO-DMT report the experience as “entering the void,” with no memory of the experience, dissociation, unresponsiveness or unconsciousness (Dourron et al., 2023; Erowid, n.d.a, n.d.b; Millière et al., 2018). Furthermore, 5MeO-DMT exposure does not evoke the immersive kaleidoscopic colours and colour shifting, or auditory distortions and hallucinations that are characteristic of “classic” psychedelics (Dourron et al., 2023; Erowid, n.d.a, n.d.b; Millière et al., 2018). 5MeO-DMT also does not benefit from extensive knowledge gained in traditional medicine and religious rituals or has been subject to the high level of recreational misuse associated with the “classic” psychedelics. From a controlled drug perspective, 5MeO-DMT is also unusual because it is not a controlled substance in the United States and Canada, but it is a C-I controlled drug in the United Kingdom and many other countries. Whether these differences will collectively persuade regulatory agencies and drug legislators to treat 5MeO-DMT and other more obscure compounds differently from the “classic” psychedelics is not yet known, but this synopsis exemplifies the complexities that will be involved in the decision-making process.

Although CDER/FDA has removed the need for an HAP trial for drug-candidates that require patients to undergo a full psychedelic experience to produce their therapeutic effect, it creates a major gap in the human data for the abuse potential assessment in the 8-Factor Analysis. To address the information deficit, CDER/FDA has recommended that drug developers should systematically record adverse events in clinical trials in far greater detail than previously and, in addition, should provide detailed patient narratives on adverse events that are closely associated with drug abuse (CDER/FDA, 2023). While there is consensus between regulatory agencies that abuse-related adverse events are an important resource for estimating the abuse risks posed by novel CNS drugs (CDER/FDA, 2023; CHMP/EMA, 2006), there may be a serious divergence of opinion on what constitutes an “adverse event.” The CDER/FDA (2023) draft guidance clearly states “*Abuse-related AEs are monitored and reported as a safety concern even if they are hypothesized to be associated with the therapeutic response.*” Hence, CDER/FDA is of the opinion that all psychoactive effects produced by psychedelic drug-candidates should be treated as adverse events because they underpin why these drugs are abused. In contrast, we are hearing that other regulatory agencies regard the psychedelic experience as a mediator of therapeutic effect, and therefore, these psychoactive effects should not be classified as adverse events.

To summarize, the psychedelics differ markedly from other CNS drugs because psychoactive events that would be considered unacceptable for the development of conventional CNS drugs, or would restrict the recommended dose range, are essential to the therapeutic actions of most psychedelic drug-candidates in late-stage clinical development. Regulatory agencies across the world are devising and implementing new strategies to meet the unique challenges associated with determining their efficacy, safety, and abuse risks. Like any process of radical change, it creates areas of divergence and uncertainty. In this review, we have identified the existing and emerging areas to be considered in the abuse potential assessment of psychedelic drug-candidates and have provided our recommendations on how they can be addressed in non-clinical and clinical development.

## The “Benzodiazepines” controlled substances scheduling model

The gamma-aminobutyric acid-A (GABA-A) positive allosteric modulators (GABA-A PAMs) comprise a large family of prescription drugs that have been developed for clinical or veterinary use, including the induction of procedural sedation, maintenance of anaesthesia, and the treatment of neurological and psychiatric disorders, for example, anxiety, panic attacks, *post-partum* depression, insomnia, epileptic seizures, and *status epilepticus*. The compounds derive from various chemical classes: the benzodiazepines, non-benzodiazepines, for example, imidazopyridine (zolpidem), pyrazolopyrimidines (zaleplon), cyclopyrrolones (zopiclone and eszopiclone), and neurosteroids, for example, brexanolone and ganaxolone. In addition to their availability as oral medications, several of them are marketed as injectable formulations, for example, diazepam, midazolam, remimazolam, and brexanolone, that pose a greater risk for diversion and abuse. The clinical pharmacology characteristics of the benzodiazepines, “Z-drugs” (zolpidem, zopiclone, and zaleplon), and neurosteroid GABA-A PAMs are summarized in Table 1. As reported in Table 1, every drug in the three sub-divisions of the GABA-A PAMs, with the solitary exception of ganaxolone, has been placed in C-IV; ganaxolone is listed in C-V.

**Table 1.** Clinical pharmacology and CSA schedules of benzodiazepines and other GABA-A receptor PAMs.

Drug name	U.S. approval year	Clinical dosing routes	Peak onset of action (hours)	Elimination half-life [active metabolite] (hours)	Therapeutic application	CSA schedule
Benzodiazepines						
Alprazolam	1981	Oral	1–3	11–13 [10–20]	Panic disorder, generalized anxiety disorder.	IV
Bromazepam	Not FDA approved. Off-label prescribing.	Oral	1–5	20–40	Severe anxiety. Hypnotic. Alcohol withdrawal	IV
Camazepam	Not FDA approved. Off-label prescribing	Oral	1–3	6–11	Anxiety	IV
Chlordiazepoxide	1960	Oral	1.5–6	36–200	Anxiety. Alcohol withdrawal. Preoperative sedation	IV
Clobazam	2011	Oral	1–5	8–60	Lennox-Gastaut syndrome	IV
Clonazepam	1975	Oral	1–5	19.5–50	Seizure disorders. Panic disorder	IV
Clorazepate	1972	Oral	0.5–2.0	[20–160]	Anxiety. Adjunctive therapy in partial seizures. Alcohol withdrawal	IV
Clotiazepam	Not FDA approved. Off-label prescribing.	Oral	1–3	4	Anxiety. Insomnia	IV
Cloxazolam	Not FDA approved. Off-label prescribing	Oral	2–5	55–77	Anxiety	IV
Delorazepam	Not FDA approved. Off-label prescribing	Oral	1–2	>79	Severe anxiety	IV
Diazepam	1963	Oral Gel Injection	1–1.5	32–205	Anxiety. Insomnia. Preoperative sedation. Alcohol withdrawal. Seizures. Muscle spasms or twitches	IV
Estazolam	1990	Oral	3–5	10–24	Insomnia	IV
Flunitrazepam	1972 Banned in USA	Oral	0.5–3	18–200	Anxiety. Insomnia	IV
Flurazepam	1970 Banned by FDA	Oral	1–1.5	40–250	Insomnia	IV
Ketazolam	Not FDA approved. Off-label prescribing	Oral	2.5–6	30–100 [36–200]	Anxiety. Insomnia	IV
Loprazolam	Not FDA approved. Off-label prescribing	Oral	2–5	6–20	Hypnotic	IV
Lorazepam	1977	Oral Injection	2–4	10–20	Panic disorder. Anxiety. Seizures	IV
Lormetazepam	Not FDA approved. Off-label prescribing	Oral	0.5–2	10–12	Anxiety. Induction of anaesthesia. Insomnia	IV
Medazepam	Not FDA approved. Off-label prescribing	Oral	4–8	36–200	Anxiety	IV
Midazolam	1985	Oral Injection Intranasal Rectal	0.5–1	1.8–6	Seizures. Anaesthesia. Anxiety	IV
Nimetazepam	Not FDA approved. Off-label prescribing	Oral	0.5–3	14–30	Anxiety. Insomnia	IV
Nitrazepam	Not FDA approved. Off-label prescribing	Oral	0.5–7	17–48	Panic disorder. Anxiety, Insomnia. Seizures	IV
Nordazepam	Not FDA approved. Off-label prescribing	Oral	1–2	30–150	Insomnia. Anxiety	IV
Oxazepam	1965	Oral	3–4	4–11	Anxiety	IV
Pinazepam	Not FDA approved. Off-label prescribing	Oral	~2	~15 [40–100]	Anxiety. Insomnia	IV
Prazepam	1976	Oral	2–6	36–200	Anxiety	IV
Remimazolam	2020	Injection		0.5–1.0	Procedural sedation	IV
Quazepam	1985	Oral	1–5	39–120	Insomnia	IV
Temazepam	1981	Oral	0.5–3	4–11	Insomnia. Preoperative sedation	IV
Tetrazepam	Not FDA approved. Off-label prescribing	Oral	1–3	3–26	Muscle spasms. Spasticity. Anxiety	IV
Triazolam	1980	Oral	0.5–2	2	Insomnia	IV
“Z-drugs” – Atypical benzodiazepine binding site ligands						
Eszopiclone	2004	Oral	~1	~6	Insomnia	IV
Zaleplon	1999	Oral	~1	~1	Insomnia	IV
Zolpidem	1992	Oral	0.5–3	2–3	Insomnia	IV
Zopiclone	Not FDA approved. Off-label prescribing	Oral	1.5–2	4–6	Insomnia	IV
Neurosteroid GABA-A receptor PAMs						
Brexanaolone	2019	Infusion	<0.25	~6	Post-partum depression	IV
Ganaxolone	2022	Oral	2–3	34	Seizures	V
Zuranolone	2023	Oral	5–6	20–25	Post-partum depression	IV

Most information sourced from the Product Labels for the drugs on the DailyMed website (https://dailymed.nlm.nih.gov/dailymed/index.cfm) supplemented by data from scientific articles. Controlled substances schedules sourced from DEA website (https://www.deadiversion.usdoj.gov/schedules/orangebook/c_cs_alpha.pdf).

CSA: Controlled Substances Act; DEA: Drug Enforcement Administration; FDA: Food and Drug Administration; GABA-A: Gamma-aminobutyric acid-A; PAMs: positive allosteric modulators.

Within the benzodiazepine class, substantial differences were known to exist between individual drugs in terms of in their potential for abuse as determined in laboratory settings (Griffiths et al., 1984; Griffiths and Wolf, 1990) and also their actual level of abuse after approval (Bergman and Griffiths, 1986; Griffiths and Wolf, 1990). In a head-to-head comparison of diazepam and oxazepam in an experienced group of recreational sedative users, diazepam produced substantially greater reinforcing and pleasurable subjective effects, for example, greater liking, peak liking, and monetary value than oxazepam (Griffiths et al., 1984). Moreover, the effects of diazepam on these measures were not only significantly greater than oxazepam at a pharmacologically equivalent 16× multiple of the clinical dose, they were also significantly greater when the clinical multiple for diazepam was decreased to 8× and 4× (Griffiths et al., 1984). The faster onset of diazepam’s pleasurable effect was favoured by this group of sedative users and cited as another reason for preferring diazepam over oxazepam (Griffiths et al., 1984). Post-marketing surveillance in Sweden revealed that in the period from 1982 to 1984 when the sales and prescriptions of diazepam and oxazepam were broadly similar, official statistics revealed the rates of prescription forgeries and mentions in theft and loss reports were approximately 250% higher for diazepam than oxazepam (Bergman and Griffiths, 1986). In a comprehensive review of the abuse potential of benzodiazepine drugs, Griffiths and Wolf (1990) considered evidence from various independent sources, that is, laboratory studies in recreational drug users, post-marketing surveillance data, interviews with experienced benzodiazepine users, and prescribing physicians, and concluded that diazepam, alprazolam, and lorazepam came with a high risk of abuse, triazolam, chlordiazepoxide, and flurazepam a moderate risk, and clonazepam, clorazepate, and oxazepam a low risk.

The safety risks of the benzodiazepines are substantially enhanced because they are frequently taken in combination with other substances of abuse, especially opiates and alcohol. It has been shown that diazepam in combination with methadone or buprenorphine evoked a greater than additive enhancement of sedation and pupil constriction and subjective opioid effects (Lintzeris et al., 2007; Preston et al., 1984), but interestingly, not drug liking (Lintzeris et al., 2007). Benzodiazepines are also extensively misused to mitigate drug withdrawal effects (Cardona-Acosta et al., 2025; Mateu-Gelabert et al., 2017; Schmitz, 2016) creating a further risk of harm from drug–drug interactions. The published evidence indicates that it is the benzodiazepines that are the more abusable identified by Griffiths and Wolf (1990), including alprazolam and diazepam which are primarily responsible.

Cardona-Acosta et al. (2025) cited statistics showing a steep increase in the misuse of benzodiazepines in combination with opioids that has emerged as part of the explosion of opioid abuse engulfing the United States. Furthermore, 70% of the synthetic opioid deaths in 2022 occurred when they were taken in combination with benzodiazepines. Alprazolam is the most widely prescribed benzodiazepine in the United States and Cardona-Acosta et al. (2025) highlighted its role in fentanyl fatalities, its prominent occurrence in emergency department visits, and its consistent ranking among the top 10 drugs involved in drug overdose deaths. Responding to the escalating safety threat posed by the misuse of benzodiazepines, in September 2020, the FDA changed the Black Box Warning on the Product Labels for the benzodiazepine drug class to highlight the serious risks and harms that can occur when benzodiazepines are taken in combination with various medicines and substances, especially opioid analgesics, alcohol, or illicit substances (FDA, 2020) (https://www.fda.gov/drugs/drug-safety-and-availability/fda-requiring-boxed-warning-updated-improve-safe-use-benzodiazepine-drug-class). This revised Black Box Warning was introduced across all benzodiazepines, but it was not applied to other GABA-A receptor PAMs, that is, Z-drug hypnotics and GABAergic neurosteroids. As discussed above, specific benzodiazepines had already been identified as posing greater public risk, but no benzodiazepine has been moved to a more restrictive CSA schedule than C-IV.

Misuse and abuse are not the only risks posed by the GABA-receptor PAMs; drug-facilitated sexual assault (DFSA) is another emerging source of serious toxicity and harm that is strongly linked with certain benzodiazepines and the Z-drugs. Flunitrazepam (Rohypnol) was the first benzodiazepine to be identified as a pharmacological weapon in DFSA. Flunitrazepam is soluble in aqueous and alcoholic drinks, and it is colourless and tasteless making it suitable for undetectably “spiking” drinks (Anglin et al., 1997; Calhoun et al., 1996; Gautam et al., 2014). Flunitrazepam has a fast onset of action, is highly sedative, induces muscular relaxation, and produces amnesia, which are added properties that make it ideal for this criminal purpose (Anglin et al., 1997; Calhoun et al., 1996; Dowd et al., 2002; Forrester, 2006; Pérez Orts et al., 2023). Numerous cases in the United States have been reported where flunitrazepam was identified as the chemical agent used in DFSA (Anglin et al., 1997; Calhoun et al., 1996; Forrester, 2006; Schwartz et al., 2000). The notoriety of flunitrazepam as a “date rape” drug led the U.S. government to pass the Drug-induced Rape Prevention and Punishment Act in 1996, and the withdrawal of flunitrazepam from the U.S. formulary, and its classification as a banned substance (https://www.congress.gov/104/plaws/publ305/PLAW-104publ305.htm). Although flunitrazepam was removed from the U.S. formulary because of its criminal use as a date rape drug, literature sources cited above reveal its continued use for this criminal act long after it was banned. Various other fast-acting benzodiazepines have also been employed in DFSA incidents, for example, alprazolam, clonazepam, diazepam, lorazepam, flurazepam, oxazepam, temazepam, and triazolam (Calhoun et al., 1996; Pérez Orts et al., 2023) along with the Z-drugs (Kintz et al., 2005; Salomone et al., 2012; Volonnino et al., 2023).

When all of findings are taken into consideration, the evidence shows that within the benzodiazepine drug class, the risk of abuse varies substantially between individual drugs. When used alone, the benzodiazepines have moderate reinforcing potential, but they are frequently taken in combination with other substances of abuse, for example, opioids, to boost the intensity and duration of the experience, which, in turn, has resulted in an upsurge in the level of benzodiazepine-related abuse and harms in the wake of the U.S. opioid abuse crisis. Finally, certain fast-acting benzodiazepines and the Z-drugs have created an additional public risk as a result of their criminal use to adulterate drinks in order to perpetrate DFSA. In spite of these document differences and changes to the patterns and risks of abuse, every benzodiazepine, Z-drug and neurosteroid GABA-A PAM with the solitary exception of ganaxolone, has been classified as a C-IV, and their restriction by controlled drug scheduling has never been revised.

**Figure 3.**
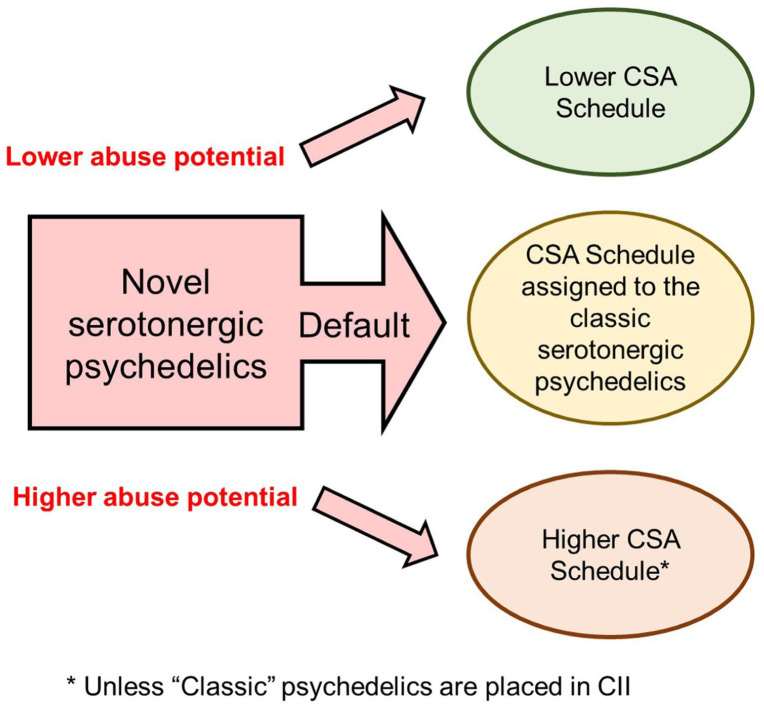
Theoretical CSA scheduling framework for classic and novel psychedelic drugs. The pharmacological mode of action of a drug is an important factor in assessing its risk of abuse. As exemplified by the GABA-A receptor PAMs, shared pharmacology can lead to shared controlled substance scheduling. 5-HT_2A_ receptor agonism is the pharmacological mechanism that is common to all conventional psychedelic drug-candidates. It is probable that the classic psychedelics and their close congeners will be allocated to the same CSA schedule. It is probable the CSA schedule assigned to the classic psychedelics will also be the default classification for the novel psychedelic drugs that follow. Although sponsors can strive to achieve lower CSA scheduling, the situation for the GABA-A receptor PAMs indicates that lower CSA scheduling will be difficult to achieve. The non-hallucinogenic 5-HT_2A_ receptor agonists are unconventional compounds with a different risk profile for abuse. CSA: Controlled Substances Act; GABA-A: aamma-aminobutyric acid-A; PAMs: positive allosteric modulators.

Why is the CSA scheduling of GABA-A PAMs relevant to the controlled drug scheduling of the psychedelics? The answer is because it illustrates that a range of drugs having a common mechanism of action, that is, potentiating GABA-A receptor signalling, has resulted in every compound under this pharmacological umbrella being placed in the same CSA schedule. This outcome has occurred despite there being meaningful differences in their potential and risk of abuse, and once the scheduling decision has been made, it is unlikely to be revisited. The conventional psychedelic drug-candidates that are in clinical development have 5-HT_2A_ receptor agonism as a common pharmacological mode of action, and in this respect, they are analogous to the GABA-A PAMs. The prediction illustrated in Figure 3 is the “classic” psychedelics and their close congeners will ultimately be placed in the same CSA schedule. This outcome has implications:

- Novel psychedelic drug-candidates will almost certainly be placed in the same CSA schedule as the “classic” psychedelics which are likely to be the first drugs to receive FDA approval.
- If the “classic” psychedelics are scheduled in C-II, newer entries to this class can strive for lower scheduling but cannot be scheduled more restrictively because C-II the highest category available for drugs with an approved medical use.
- Individual drug-candidates could be scheduled more or less restrictively than the main body of psychedelic drugs based on the demonstration of greater or lesser abuse potential and safety risks (Figure 3). The GABA-A PAMs experience teaches that the CSA schedule selected for the psychedelics is likely to include drugs with considerable differences in abuse risk, and therefore, the demonstrable risk/harm reduction required to achieve lower scheduling for a novel psychedelic drug is likely to be substantial.
- The final point of note is that the CSA schedule that is assigned to the first classic psychedelic to be approved will almost certainly set the pattern for both the early and late follower psychedelic drug-candidates.

## Non-clinical and clinical testing

Psychedelic drug-candidates fall into three broad categories, that is, “classic,” “grey area,” and “novel” (see Figure 1), and the pathway for the abuse determination and CSA scheduling of the “classic” psychedelics has been defined and publicly disclosed (CDER/FDA, 2023), (see Figure 2).

The CDER/FDA (2023) draft guidance states that “novel” psychedelic drug-candidates should have their potential for abuse evaluated according to the same principles and testing procedures as any novel CNS drug (CDER/FDA, 2017). The key information to be provided in the new drug application (NDA) and discussed in the Abuse Liability Assessment Report (the sponsor’s equivalent of the 8-Factor Analysis that is prepared by CSS) is summarized below.

1. Chemistry (includes chemical structure, ease of synthesis, aqueous solubility, and solubility in commonly available organic and inorganic solvents and ease of extraction from the marketed formulation).
2. *In vitro* receptor-ligand binding and functional studies (screening against a comprehensive panel of abuse-related molecular targets, and also a broad panel to assess the tolerability and safety risk from potential off-target interactions).
3. Pharmacokinetics in animals and humans (drugs that rapidly enter the brain pose a higher risk of abuse).
4. Abuse-related studies in animals:
  a. General behavioural observations from safety pharmacology studies (Irwin Profile and/or Functional Observation Battery (FOB)).
  b. Drug discrimination (characterization of the drug-candidate’s psychoactive properties).
  c. Self-administration (determination of the drug-candidate’s reinforcing effect).
  d. Physical dependence (relevant if the drug is intended for continuous and prolonged use).
5. Abuse-related adverse events from clinical studies (key source of information on psychoactive effects and potential for abuse).
6. Abuse-related studies in humans:
  a. HAP study (not feasible for drug-candidates that produce profound psychopharmacological effects at therapeutic doses; should be suitable to the novel “non-hallucinogenic” drug-candidates).
  b. Physical dependence study (not required for drugs that will be administered on a small number of occasions with substantial intervals between treatments. However, some are being used in clinical trials on multiple occasions separated by intervals of a few weeks. Physical dependence may also apply to novel psychedelic drug-candidates intended for continuous, prolonged use).
7. Information related to overdose, both intentional and accidental, during clinical trials.
8. Assessment of the incidence of abuse during clinical trials.

**Figure 4.**
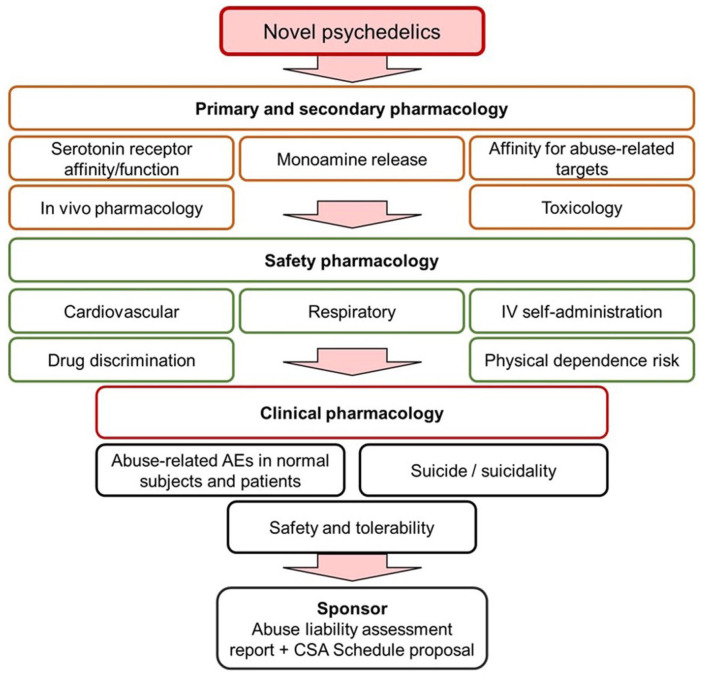
Non-clinical and clinical information required for the abuse potential assessment of novel serotonergic psychedelic drug-candidates. The FDA stated in the CDER/FDA (2023) draft guidance that novel psychedelic drug-candidates carry undefined abuse potential and safety risks and should be evaluated using the same procedures as every other novel CNS drug-candidate. This figure illustrates the non-clinical and clinical evidence required for an abuse potential assessment and scheduling determination as described by CDER/FDA (2017). If the drug-candidate produces a typical psychedelic effects, c.f., classic psychedelics, a human abuse potential study is probably not feasible. No such technical barriers exist for non-hallucinogenic 5-HT_2A_ receptor agonists. CDER: Center for Drug Evaluation and Research; CNS: central nervous system; FDA: Food and Drug Administration.

In Figure 4, we propose a screening cascade suitable for gathering the information required to assess the abuse, dependence, and safety risks associated with “novel” psychedelic compounds. In this review, we limit the discussion to a brief summary of the non-clinical testing procedures, because we have previously published comprehensive reviews on these topics (Heal et al., 2018, 2023; Henningfield et al., 2023). We have, however, provided an update on developments in the field that have influenced or changed the opinions offered in these earlier publications. In addition, we have extended the discussion to cover the latest approaches in psychedelic drug development that are being adopted by pharmaceutical and biotech companies.

## Non-clinical testing

### Chemistry

The first level of *in silico* screening in the drug abuse assessment process is to determine if the drug-candidate’s chemical structure is related to that of any controlled substance of abuse. For novel psychedelics, it is highly likely that there will be structural similarities to existing drugs, especially as many of them may have been used as chemical templates for synthesizing the clinical candidate molecule. In addition to chemical structure, the ease of synthesis of the drug-candidate, its aqueous solubility, solubility in commonly available organic and inorganic solvents, and ease of extraction from the marketed formulation are all taken into account when estimating the risk of abuse (Heal et al., 2018, 2023).

### Receptor-ligand binding studies and functional studies *in vitro*

*In vitro* screening is conducted to define the affinity and function of the drug-candidate on 5-HT_2A_ and other 5-HT receptor subtypes as part of defining the drug-candidate’s mode of action and to help predict tolerability and safety.

Many of the psychedelic compounds are not “clean” molecules and come with a multiplicity of pharmacological actions that could have implications for efficacy and safety. *In vitro* screening will also investigate whether the drug-candidate has affinity for a comprehensive panel of molecular targets that mediate the actions of known substances of abuse. The targets include dopamine, serotonin (e.g., 5-HT_2A_ and 5-HT_2C_), γ-aminobutyric acid (GABA)/benzodiazepine, opioid (mu, kappa, and delta), cannabinoid (CB_1_ and CB_2_), nicotinic, NMDA, and orexin receptors, as well as ion-channel complexes and transporter sites (e.g., serotonin and dopamine). For “classic,” “grey area,” and “novel” psychedelics, it is assumed that 5-HT_2A_ receptor agonism will be an essential component of its therapeutic effect. *In vitro* screening is also informative because there is ongoing speculation that other pharmacological components of the “classic” psychedelics may be of therapeutic importance. Finally, it is probable that psychedelic compound may also have pharmacologically relevant affinity at other abuse-related molecular target(s). If that is the case, the drug-candidate’s affinity and functional activity should be determined for these other abuse-related molecular targets (Heal et al., 2018, 2023).

*In vitro* screening should also be performed to test the binding affinity of the test substance against a wide panel of potential off-targets (e.g., receptors, modulatory binding sites, ion channels, transporters, and enzymes) additional to those that are abuse-related. This is an important step to evaluate the tolerability and safety risk from potential off-target interactions. Although the psychedelics in late-stage development are administered to patients in one or two supervised sessions, some early-stage “novel” psychedelics are being developed for continuous long-term use. It has been established that 5-HT_2B_ agonists can cause cardiac valve damage (Rothman et al., 2000; Setola et al., 2005), and the majority of 5-HT_2A_ receptor agonists, for example, psilocin, mescaline, DMT, 5MeO-DMT, LSD, DOI, and DOB, are 5-HT_2B_ receptor partial agonists. For psychedelics administered once or twice, cardiac damage is a non-issue. Novel “psychedelics” for continuous use come with a risk of cardiac valve damage on repeated and prolonged exposure, and there is always the prospect of individuals misusing or microdosing-approved psychedelic drugs to self-medicate perceived or real medical conditions.

### Pharmacokinetics in animals and humans

The pharmacokinetic profiling to determine absorption, distribution, metabolism, and excretion (ADME) of a test substance is a crucial factor in its safety assessment during early drug development. Differences in the pharmacokinetics of a test substance may indicate the different abuse liability profiles of drugs arising from the same pharmacological class.

Subjects that abuse drugs intend to achieve the desired effect as rapidly as possible after taking it. Hence, drugs with abuse potential that rapidly enter the brain are associated with higher potential for abuse. To achieve the maximum effect, abusers employ inhalation or intravenous injection because they are routes of administration that are associated with a rapid absorption and a fast onset of effect. Rapid absorption rate and high lipid solubility are the most important factors contributing to early drug concentrations in the brain. The other pharmacokinetic properties that contribute to increased self-administration and abuse of a drug include high bioavailability, short half-life, low protein binding, and small volume of distribution. These are important factors in the early screening of drug-candidates for their abuse potential.

### Abuse-related studies in animals: General behavioural observations from safety pharmacology studies – Irwin Profile and FOB

The Irwin Profile assesses the CNS effects of drug-candidates using a series of behavioural and motor assessments in rats. A wide spectrum of doses is evaluated from those which are pharmacologically relevant to those in the toxicological range. Behavioural signals of 5-HT_2A_ receptor agonism, including the induction of head-twitches or wet dog shakes should be monitored when reporting CNS effects (Heal et al., 2018). The Irwin Profile also determines the general characteristics of the test substances such as inducing sedation, stimulation, or behaviourally neutral effects.

Profiling of a drugs behavioural and physical effects can also be performed using an FOB, often carried out as part of drug toxicity studies. The FOB is a procedure that is carried out to detect gross functional deficits in rats resulting from exposure to a test substance. This battery of tests is designed to be used together with neuropathologic evaluation and/or general toxicity testing. Following test substance administration, animals are observed under controlled conditions at regular intervals to monitor for any behavioural and neurologic abnormalities (Gauvin and Baird, 2008).

### Head-twitch behaviour

Head-twitches in mice and wet dog shakes in rats are widely used as behavioural models for detecting 5-HT_2A_ receptor agonist activity and “psychedelic” properties. Evidence in support of this hypothesis comes from the strong correlation between the potency of phenylalkylamine and tryptamine 5-HT_2A_ receptor agonists to induce head-twitches and induce hallucinations in humans (Halberstadt et al., 2020). This response is inhibited by the highly selective 5-HT_2A_ antagonists (Green and Heal, 1985; Heal et al., 1992; Jennings et al., 2008; Willins and Meltzer, 1997), and has been shown to be abolished in 5-HT_2A_ receptor knockout animals (Gatch et al., 2013; González-Maeso et al., 2007).

When used within its limitations, the model is a fast and easy screen. Thus, head-twitches are induced by 5-HT_2A_ agonist hallucinogens, including LSD, mescaline, psilocybin, and N,N-DMT, and novel synthetic 5-HT_2A_ receptor agonists, for example, 25I-NBOMe (Kueppers and Cooke, 2015; Rose et al., 2013) and 25I-NBOH (Contreras et al., 2021; Halberstadt and Geyer, 2014). The model’s limitations are that head-twitches can be elicited by hallucinogens from other pharmacological classes, for example, PCP and ketamine, and by a wide range of non-hallucinogenic drugs that potentiate serotonergic function, for example, 5-hydroxytryptophan (5-HTP), fenfluramine, quipazine, p-chlorophenylalanine (PCPA) (Bedard and Pycock,1977; Corne and Pickering, 1967; Corne et al.,1963; Green and Heal, 1985; Heal et al., 1992). Finally, it is important to appreciate that head-twitch behaviour is markedly inhibited by drugs that also act on dopamine, noradrenaline and GABA neurotransmitter systems (Green and Heal, 1985; Heal et al., 1992) which creates the possibility that psychedelic drugs which have complex pharmacology can show up as “false negatives” in this test.

### Drug-discrimination: Characterization of the drug-candidate’s psychoactive properties

Drug discrimination is performed to determine whether the psychoactive effects of the drug-candidate are identical, or similar, to those of known substances of abuse. In safety pharmacology, drug discrimination is performed at doses of the drug-candidate that generate plasma *C*_max_ concentrations in animals that are equivalent to those in humans after the therapeutic dose and two to three fold higher to cover doses that are often abused (CDER/FDA, 2017).

In previous reviews, we have comprehensively discussed the use of the drug discrimination both as a model to detect the compounds with psychedelic properties for clinical development and as a safety pharmacology screen for assessing abuse potential (Heal et al., 2018, 2023). The discriminative cue that animals are trained to recognize is pharmacologically specific, and it is both a strength and a weakness of the model (Heal et al., 2025). For this reason, we advocate the use of selective 5-HT_2A/2C_ receptor agonists, DOI, DOB (2,5-dimethoxy-4-bromoamphetamine), or DOM as the training stimulus in this test. The serotonergic hallucinogens, DOI, DOB, and DOM produce a discriminative cue in animals that is predominantly mediated by 5-HT_2A_ receptor partial agonism with a minor contribution via 5-HT_2C_ receptors (Chojnacka-Wójcik and Kłodzińska, 1997; Ismaiel et al., 1993; Schreiber et al., 1994; Smith et al., 2003). Many serotonergic hallucinogens have been reported to generalize to DOI, DOB, and DOM (Eshleman et al., 2014; Glennon et al., 1983; Halberstatdt et al., 2020). In contrast, when LSD is employed as the training stimulus, it generates a complex interoceptive cue. As an example, LSD’s cue is not only blocked by the selective 5-HT_2A_ antagonists, ketanserin, pirenperone, ritanserin, and MDL 100,907 (Arnt and Hyttel, 1989; Colpaert et al., 1982; Cunningham and Appel, 1987; Goodwin et al., 2003; Gresch et al., 2007), but it is also partially inhibited by dopamine D_4_ antagonists (Marona-Lewicka et al., 2009) and mGlu(2/3) agonists (Winter et al., 2004). The implication is that the greater the pharmacological complexity of the stimulus cue, the greater the opportunity to generate false positive or false negative results in safety pharmacology testing.

Several companies are actively exploring the therapeutic potential of 5MeO-DMT as a treatment for a range of psychiatric disorders. In view of its atypical pharmacological and psychoactive profile, we have suggested that 5MeO-DMT could be a candidate for inclusion in the “grey area” psychedelics. 5MeO-DMT is naturally occurring psychedelic, which is atypical because it is a high affinity, full agonist at both 5-HT_2A_ (EC_50_ = 26 nM) and 5-HT_1A_ (EC_50_ = 25 nM) receptors (Warren et al., 2024). Earlier classification of 5MeO-DMT as a mixed 5-HT receptor subtype ligand with low micromolar affinity for the 5-HT_2A_ receptor was based on its displacement of antagonist radioligands in binding experiments (Glennon et al., 1980; Halberstadt et al., 2012; Ray, 2010) that misled the field for many years, and is incompatible with the compound’s potent 5-HT_2A_ agonist effects in animals (Green and Heal, 1985; Heal et al., 1985, 1986, 1992; Metz and Heal, 1986) and in humans (Davis et al., 2018; Reckweg et al., 2023; Rucker et al., 2024; Weil and Davis, 1994).

5MeO-DMT’s mixed 5-HT_2A_ /5-HT_1A_ agonist pharmacology has generated inconsistent results and contradictory hypotheses concerning the serotonergic mechanism(s) mediating its stimulus cue. Since drug discrimination is a key test in abuse potential evaluation, it is important to have clear insight into 5MeO-DMT’s interoceptive cue, and its implications when conducting drug discrimination testing on novel drug-candidates with 5-HT_2A_ /5-HT_1A_ full agonist properties. From our analysis of the published data, we conclude that 5MeO-DMT generates an interoceptive cue which has substantial contributions from its 5-HT_2A_ and 5-HT_1A_ agonist properties. Thus, it has been reliably shown that DOM and LSD fully cross-substitute with the 5MeO-DMT cue (Arnt, 1989; Glennon et al., 1979; Spencer et al., 1987; Young et al., 1982), and full cross-substitution also occurs between 5MeO-DMT and a range of highly selective 5-HT_1A_ agonists (Arnt, 1989; Schreiber and de Vry, 1993; Spencer et al., 1987; Winter et al., 2000). Antagonist characterization of 5MeO-DMT’s stimulus cue revealed that the greatest inhibition was achieved using non-selective 5-HT_1_/5-HT_2_ receptor antagonists, for example, pizotifen and methiothepin (Glennon et al., 1979; Spencer et al., 1987; Young et al., 1982), whereas selective 5-HT_1A_ antagonists, for example, WAY 100,635, pindolol, and 5-HT_2A_ antagonists, for example, pirenperone, and ketanserin, produced partial inhibition or rightward shifts in the 5MeO-DMT dose-response function (Spencer et al., 1987; Winter et al., 2000). What about the anomalies? There are inconsistencies in the results, and it has to be conceded that there are no convenient explanations for some of them. From our reading of the literature, it appears the level of generalization against other stimulus cues that can be achieved with 5MeO-DMT was often limited by the onset of the serotonin syndrome which blocked operant responding in the drug discrimination test. Marked inhibition of operant responding by other serotonergic agonists and antagonists is also a likely confounder in many of the studies. The implication is drug-discrimination testing using a 5-HT_2A_ receptor agonist, for example, DOM or LSD, as the training cue could produce a false negative result when evaluating novel drugs which are potent 5-HT_2A_/5-HT_1A_ full agonists. As a precaution, therefore, we suggest that a drug-candidate with this pharmacological profile should also be characterized in a 5MeO-DMT-cued drug-discrimination model.

### Self-administration: Determination of reinforcing effect

Animal self-administration studies have excellent validity in predicting the relative abuse potential of a drug. Many classes of drugs used for recreational purposes such as cocaine or heroin that have rewarding properties in humans also produce rewarding psychoactive effects in animals. A comprehensive overview of intravenous self-administration as a safety pharmacology screen for assessing abuse potential of psychedelic compounds is provided two earlier reviews (Heal et al., 2018, 2023).

In the review by Heal et al. (2018), we reported that the reinforcing potential of several psychedelic 5-HT_2A_ receptor agonists had been investigated in intravenous self-administration experiments, but none had shown efficacy (Fantegrossi et al., 2004; Goodwin, 2016; Yanagita, 1986). It could be animals do not experience the somatic, sensory and perceptual changes produced by the psychedelics as rewarding, or that rapid pharmacological tolerance extinguishes responding before a stable level of self-administration can be achieved. Whatever the reason, it is probable that most, if not all, conventional 5-HT_2A_ agonist psychedelics will not serve as positive reinforcers in the intravenous self-administration model. On the other hand, novel compounds that combine 5-HT_2A_ agonism with monoamine releasing activity, c.f., 25B-NBOMe and various cathinones, are self-administered by rats (Creehan et al., 2015; Custodio et al., 2020) so the model still has an important role to play in the abuse potential evaluation of novel 5-HT_2A_ agonist drug-candidates. The emergence of 5-HT_2A_ agonists that are devoid of psychedelic or hallucinogenic properties comprises another group of drug-candidates is another factor for consideration. These compounds differ from conventional psychedelics because they are intended for continuous daily dosing rather than administration in one or two medically supervised sessions. Given their very different psychoactive properties, these non-hallucinogenic 5-HT_2A_ agonists may have positive reinforcing effects, and therefore, require testing in the intravenous self-administration model.

As a general rule, psychedelic drug-candidates are likely to possess weak to moderate reinforcing effects, and therefore, the experimental conditions should be set to ensure the model has the relevant sensitivity to detect an abuse signal. Rats will not acquire and maintain self-administration of weak reinforcers (Heal et al., 2018, 2023). They first need to be trained using a strong reinforcer, for example, heroin or cocaine, on a low fixed ratio reward schedule. After saline extinction (to eliminate false responders), the psychedelic drug-candidate can then be substituted in the model to determine whether it has positive reinforcing effects. Given 5-HT_2A_ receptor agonists produce powerful psychoactive effects,

individual doses of the drug-candidate should be used to avoid false negative outcomes due to over-dose occurring, or satiation from a low number of high dose infusions of the drug-candidate (CDER/FDA, 2017; Heal et al., 2018, 2023).

### Physical dependence

For a psychedelic drug-candidate that will be administered to patients on one or two occasions under medical supervision, and possibly at intervals separated by months or years thereafter, the likelihood of a patient becoming physically dependent is vanishingly small. Hence, a non-clinical assessment of the drug-candidate’s liability to produce physical dependence on withdrawal would not be a relevant requirement for “novel” psychedelics employing this type of treatment regimen. However, conventional psychedelic drug-candidates intended for continuous daily use at low doses, or novel non-hallucinogenic 5-HT_2A_ agonists that employ a similar dosing regimen, will have been required to undergo non-clinical testing to investigate their potential to induce pharmacological tolerance and physical dependence on withdrawal.

Of itself, physical dependence is not considered to be an abuse potential signal because it is a physiological state of adaptation in response to repeated drug use and can occur in response to many CNS drugs that have no abuse liability, for example, SSRIs. Although it has long been known that the 5-HT_2A_ receptor in animals and human brain exhibits rapid desensitization on repeated exposure to agonists (Abramson et al., 1956; Cholden et al., 1955; Darmani et al., 1992; Isbell et al., 1959; Leysen et al., 1989; Schindler et al., 2012), they are not believed to produce physical or psychological dependence on drug withdrawal. Moreover, we could find no reports of psychedelic drugs including directly and indirectly acting 5-HT_2A_ receptor agonists causing withdrawal induced physical dependence.

The physical dependence test is part of the abuse and dependence potential assessment that is required by regulatory authorities (CDER/FDA, 2017; CHMP/EMA, 2006). Its application to evaluating psychedelic drug-candidates is described and reviewed in Heal et al. (2018) and Heal et al. (2023).

## Clinical investigations

Information on CNS drug-candidates obtained from drug-experienced volunteers in an HAP trial has a prominent role in the FDA assessment of abuse potential. Most CNS drugs are prescribed at doses which may elicit psychoactive adverse events, but they are generally at a low to moderate level of severity and often disappear after a few days of treatment. The abuse potential assessment in an HAP trial is conducted at the therapeutic dose level and 2–3× higher, which is the dose range where the drug is likely to be abused. The therapeutic effects of the classic and novel conventional psychedelic drug-candidates are thought to derive from a fully immersive psychedelic experience (although this is a matter of ongoing debate and research). Hence, it precludes any objective collection of data from an HAP trial because of the difficulty of “blinding” the sessions or employing a placebo control to benchmark the findings. In addition, there are no very suitable controlled drugs in C-II to C-V that can be used as a positive control.

Although it is not feasible to extract reliable and objective data for a classic or conventional psychedelic drug-candidate from an HAP trial, these restrictions will not apply to novel, non-hallucinogenic psychedelics. As stated in the draft guidance released by CDER/FDA (2023), these “novel” psychedelics will be treated like any other CNS drug-candidate which implies that if abuse signals are observed in non-clinical or clinical investigations, the drug-candidate will probably be required to undergo evaluation in an HAP study. The choice of positive control and suitable population of drug-experienced volunteers for the trial will need to align with the principles set out in the CDER/FDA (2017) guidance. The positive control should be selected based on the similarities between the psychoactive effects evoked by drug-candidate and known drugs of abuse and the intended therapeutic application. The positive control could be an approved drug in C-II to C-V, but in reality, it is likely to be selected from C-II to C-IV. There are two reasons: first, the positive control needs to produce ⩾15 point difference relative to placebo on the primary endpoint of *E*_max_ Drug Liking *at this moment* to validate the study and C-V drugs lack the euphoriant efficacy to deliver this outcome, and second, scientists from CSS now believe that several approved dual orexin antagonists and anticonvulsant medications that had been assigned to C-V failed to show evidence of abuse following approval suggesting they are probably not substances of abuse (Calderon et al., 2020; Caro et al., 2022). Although pharmacological mechanism of action is theoretically a determinant when selecting the positive control for an HAP study, it is difficult to envisage a conventional psychedelic 5-HT_2A_ agonist being selected, even after approval and CSA scheduling, for the technical reasons stated above.

The inability to perform HAP trials on the conventional psychedelics has prompted a shift toward the abuse potential assessment model adopted by EMA (CHMP/EMA, 2006), which does not require evidence from specialized trials in drug-experienced volunteers. The human experience is pivotal in the abuse potential assessment and, therefore, systematically reporting psychoactive effects and emotional responses during the psychedelic session and recording adverse events that are suggestive of an abuse risk (irrespective of whether or not they are mediators of efficacy) is the only viable approach for accessing this critical information. The FDA’s more intensive approach to collecting, interpreting, and reporting abuse-related adverse events was presented Dr Steven Galati at the Cross-Company Abuse Liability Council (C-CALC) Meeting in 2023 (Galati, 2023), and his highly informative slides can be accessed from the C-CALC website (https://img1.wsimg.com/blobby/go/933911af-c640-4678-bef3-07ff76ae9c47/downloads/Topic%203%20%20AEs%20and%20Analysis_Steven%20Galati%20FDA.pdf?ver=1705683910630). The adverse events reported by patients (“intended population” (individuals who are taking the drug to treat their medical disorder)), and healthy volunteers (“unintended population” (individuals who have not been prescribed the drug to treat a disease or disorder)) represent opinions of two very different populations. Separate analysis offers insights relevant to the abuse risk to patients and the general public. Although healthy volunteers with no predisposition to abuse drugs lack the element of susceptibility provided by experienced recreational drug users, nevertheless, the information they provide is a workable substitute. A detailed discussion on this topic is outside of the scope of our review. For a comprehensive exposition, readers are directed to the slide presentation by Galati (2023), which provides clear guidance to drug developers on the revised and extended list of adverse events FDA considers indicative of abuse, the classification of adverse events into low and high level categories (the latter requiring physician narratives), and FDA’s expectations on reporting of the findings in the NDA and in the Abuse Liability Assessment Report.

The sponsor is also required to provide information about the consequences and sequelae of accidental or intentional overdose of the drug-candidate; the issue of intentional overdose is inextricably linked with suicidal ideation, motivation, and unsuccessful or successful attempts at suicide. Because the classic and conventional psychedelics are administered by a physician in a controlled setting, the risk of an accidental overdose is remote, and there is no opportunity for an intentional overdose. Nevertheless, suicidal ideation, motivation, and suicide are an important component of the risks associated with the abuse of classic and novel psychedelic drug-candidates.

Although the number of indications being explored as targets for psychedelic intervention is continuously expanding, the majority of drug development and clinical trials are aimed at the treatment of psychiatric disorders, for example, major depressive disorder, treatment resistant depression (TRD), anxiety/depression in terminal illness, and post-traumatic stress disorder. Meta-analyses have consistently revealed a significantly increased relative risk of suicide in individuals with mental disorders (Le et al., 2024; Moitra et al., 2021; Sutar et al., 2023; Too et al., 2019). The literature on a possible link between suicide and the non-medical use of psychedelics paints a confusing picture with reports of increased, decreased, and no change in the suicide risk associated with psychedelic use (Zeifman et al., 2021). In the Phase 2 trial of psilocybin in TRD, suicidal ideation (passive or active but with no intent or plan) was reported in approximately 30% of the subjects in all treatment groups at baseline, but to confuse the issue, there were also three reports of serious adverse events of suicidal ideation in subjects who had been administered both the clinically effective (25 mg) and ineffective (10 mg) doses of psilocybin (Goodwin et al., 2022). Zeifman et al. (2022) recently published a meta-analysis which suggested that results from recent clinical trials with psychedelic drugs indicate they reduce suicidal ideation and the risk of suicide, whereas Meshkat et al. (2025) reported that the benefits of psychedelic interventions remain inconclusive. With no consensus on the issue, taking into account the vulnerability of many of the patient groups being treated with psychedelic drug-candidates, close monitoring, and reporting of suicidal ideation and suicide attempts are an essential part of the assessment of the possible risks associated with the abuse of these novel drugs.

Once a drug-candidate’s potential for abuse has been established in non-clinical and clinical studies, safety in overdose and drug-induced toxicity are key factors when estimating relative risk before assigning it to a CSA schedule. Returning to the regulatory history of the benzodiazepines for a moment, these drugs were hailed as safe and effective alternatives to the barbiturates because the risk of an overdose-related fatality was greatly reduced when compared with the barbiturates that they largely replaced. In contrast to the benzodiazepines and GABA-A receptor PAMs, which are almost universally in C-IV, due to their widely varying risks and harms, there are barbiturates in C-II, for example, pentobarbital and secobarbital, C-III, for example, aprobarbital, thiopental, and vinbarbital, and C-IV, for example, methohexital and phenobarbital. In crude terms, drugs that carry a high risk of abuse and limited headroom between the quantities that individuals are likely to abuse and an overdose requiring an emergency room visit and/or a fatality are likely to be assigned C-II scheduling. Among the tragic statistics documenting U.S. drug overdose deaths, the greatest culprits are the μ-opioid receptor agonists, for example, fentanyl and oxycodone, and stimulants, for example, methamphetamine and cocaine; unsurprisingly, these clinically approved drugs are all in C-II. Thus, the sponsor is advised to diligently and systematically collect and report adverse events from patients and volunteers that may occur when the recommended dose is exceeded, the severity of the harms, and the likelihood of prolonged disability or death. Collectively, the findings can be used to calculate the multiple of the recommended dose that can be taken without risk of serious harm, which is essential information in decision-making for controlled drug scheduling.

**Figure 5.**
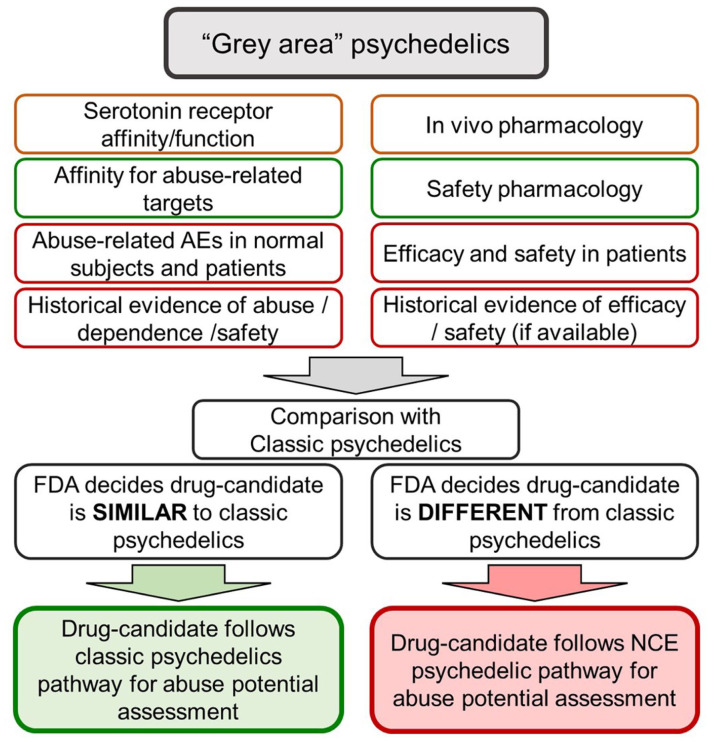
Non-clinical and clinical evidence to determine appropriate regulatory pathway to evaluate the abuse potential assessment of “grey area” psychedelic drug-candidates. Grey area psychedelics consist of compounds with less comprehensive data on abuse potential and safety than novel molecules which are close analogues of the classic compounds. The route for abuse potential assessment of the grey area psychedelic drug-candidates is not clear. We have recommended the package of non-clinical and clinical evidence to be collected and analysed to assess which developmental path is appropriate for the drug-candidate. The sponsor should collect and evaluate the data from these non-clinical and clinical categories and submit the evidence along with its proposal for the abuse potential evaluation pathway to the FDA for a definitive decision on the matter. FDA: Food and Drug Administration.

Figure 5 illustrates the regulatory pathway for assessing the abuse and dependence potential of drug-candidates that reside in the “grey area” category. These compounds require an initial evaluation to collate the non-clinical and clinical evidence shown in the upper section of Figure 5 to determine which of the alternative development pathways is appropriate. In the case of known psychedelics with limited human experience, the information may include published or anecdotal evidence on abuse, dependence and associated risks/harms, and/or therapeutic efficacy and safety. The non-clinical and clinical evidence for the “grey area” drug-candidate should be compared/contrasted against the mirror image data for the “classic” psychedelic molecule used to model its abuse and dependence risks. The objective is to demonstrate that although the two compounds are not identical, the pharmacology, abuse, dependence, and safety/tolerability risks of the “grey area” drug-candidate are sufficiently similar to those of a specific “classic” psychedelic; the latter can be used as a surrogate to predict the risks of the former. The formal case should be submitted to the FDA for a decision. If the FDA accepts the sponsor’s proposal that there is adequate similarity between the “grey area” drug-candidate and the selected “classic” psychedelic, it will follow the development path described in Figure 2. Thus, there would be no requirement to evaluate the “grey area” drug-candidate in the standard safety pharmacology package of non-clinical abuse and dependence tests. It does not remove the obligation of the sponsor to collect clinical data on psychoactive effects (including those associated with abuse or dependence), safety, tolerability, and adverse events on the “grey area” drug-candidate. Given that the abuse/dependence risks of the “grey area” drug-candidate are considered by the FDA to be identical to those of the “classic” psychedelic, it follows that if both compounds are approved for medical use, they will ultimately be placed in the same CSA schedule. Alternatively, if the FDA does not accept, there is sufficient similarity between the “grey area” drug-candidate and the selected “classic” psychedelic compound; the former will be classed as a “novel” psychedelic and follow the conventional abuse and dependence assessment pathway illustrated in Figure 4.

Irrespective of which regulatory pathway is taken by the psychedelic drug-candidate, the non-clinical and clinical evidence to evaluate the abuse and dependence risks posed by novel psychedelic drug-candidates will be collected, integrated, and submitted as part of the NDA in the USA, Marketing Authorization Application in the United Kingdom and Europe, and their equivalents elsewhere in the world. The mechanism by which the sponsor and regulatory agency will review and assess the information collected on a psychedelic drug-candidate is discussed in the following section.

## The 8-factor analysis

As illustrated in Figures 2 and 4, irrespective of the type of psychedelic drug-candidate, the non-clinical and clinical findings relevant to abuse, dependence, and their associated tolerability, safety, and toxicity risks need to be collated and evaluated as part of the drug approval submission. This sponsor’s abuse potential evaluation of the drug-candidate together with a proposal on controlled drug scheduling has to be provided to the regulatory agency. The procedures vary from country to country, and we have previously compared and contrasted the United States and European regulatory procedures and the information required for decision-making procedures (Calderon et al., 2015). Certain principles apply irrespective of where the drug-candidate is seeking approval.

- Controlled drug scheduling (or not) is a product labelling matter that is separate from the benefit/risk evaluation that underpins the decision on whether to approve the drug for medical use in humans.
- Controlled drug scheduling is a relative, not an absolute, judgement; thus, a recommendation for C-IV scheduling should be benchmarked by showing that the evidence predicts that the drug has a lower abuse risk than substances in C-III and a greater risk than those in C-V.
- If the drug-candidate has not previously been medically approved in any other country, controlled drug scheduling is a “best guess” that may, or may not, prove to be accurate when the drug appears in pharmacies, hospitals, and is in widespread clinical use.

Many of the “classic” and some of the potential “grey area” psychedelic compounds are unusual because they are well-known compounds, they have been classified as C-I controlled drugs, and there are decades to millennia of human experience on which to determine their potential for abuse. As concluded by the FDA, there is ample evidence available to assign scheduling under the CSA (CDER/FDA, 2023), and the element of guesswork will be very low.

The 8-Factor Analysis performed by the FDA and its equivalent, that is, the Abuse Liability Assessment Report, which is prepared by the sponsor, is a highly structured framework that uses a constant set of parameters to evaluate every CNS drug, thereby ensuring the determination of relative risk of abuse and CSA scheduling is robust, consistent, and reliable.

The assessment parameters in the 8-Factor Analysis are as follows.

### Factor 1: Its actual or relative potential for abuse

This factor is key to the abuse potential assessment because it contains all of the non-clinical and clinical information relevant to assessing its potential for abuse. Results from the *in vitro* screening of psychedelic drug-candidates against a broad range of abuse-related and safety CNS targets are relevant because pharmacological interactions in addition to 5-HT_2A_ receptor agonism may be relevant, for example, 5MeO-DMT, and influence the potential for abuse. As shown in Figure 4, findings from the non-clinical abuse/dependence safety pharmacology package that have been generated with “novel” psychedelics will be included in Factor 1, whereas for “classic” psychedelics, information on this topic can be sourced from publications. The systematic description of events occurring with psychoactive experience in clinical trials, combined with a comprehensive analysis of the spectrum of abuse-related adverse events, including their frequency and severity, will form the foundation of abuse potential assessment (CDER/FDA, 2023). Psychedelic drugs are being developed to be used clinically in single sessions or a few sessions spread over weeks or months. This treatment regimen reduces the level of non-clinical toxicity and safety testing required for registration of the new drug. It also addresses the safety risks to patients administered these drugs under medical supervision according to the Product Label or Summary of Product Characteristics. However, it does not cover risks to those individuals who self-medicate, misuse or abuse psychedelic drugs. In the case of the “classic” psychedelics, the clinical study evidence will be supplemented by data from human studies and statistics about the extent of human misuse/abuse and their associated harms sourced from current and historical databases, for example, Treatment Episode Data Set, National Survey on Drug Use and Health, and the Drug Abuse Warning Network.

### Factor 2: Scientific evidence of its pharmacological effect, if known

Based on a wealth of non-clinical and clinical sources, there is strong evidence that 5-HT_2A_ receptor agonism is the mediator of the emotional, perceptual, and sensory experiences produced by conventional psychedelics (Glennon et al., 1984; Holze et al., 2020; Preller et al., 2017, 2018, 2019; Titeler et al., 1988). The majority view is that the psychedelic experience is the source of the therapeutic effects (Kangaslampi, 2023; Ko et al., 2022; Romeo et al., 2025), though there are dissenting views (Olson, 2020). What is unclear for most psychedelics is the contribution of the drug-candidate’s secondary pharmacological actions to the onset, magnitude, and maintenance of therapeutic effect; if known, they should be reported and discussed in this factor.

### Factor 3: The state of current scientific knowledge regarding the drug or other substance

This section includes evidence relating to ADME; pharmacokinetics; toxicology and safety pharmacology; drug-drug interactions; and tolerability, safety, and adverse events in humans. This information offers a wider perspective about the drug-candidate’s risks and harms. For “classic” psychedelics and drug-candidates that FDA has deemed to be sufficiently similar for them to share a common abuse potential risk, this information can be supplemented with information from published sources.

### Factor 4: Its history and current pattern of abuse

If the drug-candidate falls into the “classic” psychedelics category, information on this topic can be from publications and recognized safety databases. For “novel” psychedelics, the predictions on “classic” psychedelic drugs carry far less evidential weight and should be interpreted with caution.

### Factor 5: The scope, duration, and significance of abuse

The same caveats apply here. Although the scope, duration, and significance of abuse is well understood for the “classic” psychedelic and drug-candidates with similar pharmacology, the predicted risks may be inaccurate when dealing with “novel” psychedelic drug-candidates. It is very likely that the abuse potential risks posed by non-hallucinogenic 5-HT_2A_ receptor agonists will be very different from the conventional psychedelics and quite probably much lower; on the other hand, as exemplified by the NBOMe’s (N-methoxybenzyl-group of psychedelics), these substances pose a far greater risks than the “classic” psychedelics.

### Factor 6: What, if any, risk there is to the public health

The term “public” in this case comprises two separate groups, that is, patients who will be prescribed the drug under medical supervision (the “intended population”) and individuals who misuse or abuse the drug for experimentation, recreation or self-medication (the “unintended population”). The risk to patients of abuse and/or dependence is reflected in the Product Label, for example, Warnings or Precautions about prescribing to patients with a history of substance abuse, or a limitation on the duration of treatment in the case of physical or psychological dependence. It is the responsibility of the relevant FDA therapeutic area division, for example, Division of Psychiatry, Division of Anaesthesiology, Addiction Medicine, and Pain Medicine, or the Divisions of Neurology I and II, to conduct the benefit/risk assessment and decide on approval. Although the CSS Division conducts the abuse potential assessment that covers potential areas of misuse and abuse outside of the approved medical use, it will also advise the appropriate therapeutic area division on matters relating to abuse and/or dependence relevant to patients. The CSS Division will also provide advice on the framing of the Product Label. There is ample evidence to define and quantify the risks to the public health (intended and unintended populations) posed by the “classic” psychedelic, and drug-candidates with similar pharmacology. In addition to the risks to individuals who abuse the drug to experience its psychedelic effects, there is the potential threat from its misuse in the pursuit of personal enlightenment, well-being, and self-medication (Garel et al., 2024; Myran et al., 2025). The increase in reports of hospitalizations and adverse events associated with the experimentation and misuse of psychedelics (Garel et al., 2024; Honk et al., 2024; Kopra et al., 2025; Lebedev et al., 2021; Malcolm and Thomas, 2022; Wilkes et al., 2024) is unfortunately an inevitable consequence of the feverish interest in the psychedelics that fills social networks and the media. The potential risks and harms to public health arising from the widespread use of the psychedelics will be a factor that is given considerable prominence in any decision on controlled drug scheduling. That said, the discussion of the risk to public health should not be limited to the drug-candidate’s potential harms but should include a description of the health benefits that will accrue from introducing a clinically effective treatment for the disorder or disease. Alleviating disability brings great benefit to patients, their families, and caregivers; it reduces the cost and resources burden on the healthcare system and hopefully allows the patient to return to a situation of contributing to society.

### Factor 7: Its psychic or physiological dependence liability

“Classic” and “novel” drug-candidates that elicit their therapeutic effect through participation in a conventional psychedelic experience are intended for use in one or two treatment sessions with *ad hoc* follow-up sessions to maintain efficacy and prevent relapse. The possibility of a drug producing physical or psychological dependence when administered according to this dosing regimen is minimal. In contrast, the possibility of emerging physical or psychological dependence is a serious consideration for a drug-candidate that is intended for continuous daily dosing or administration at frequent intervals. These risks should have been assessed during the non-clinical and clinical development of the drug-candidate and the findings and their implications for patient and public safety discussed in this factor.

### Factor 8: Whether the substance is an immediate precursor of a substance already controlled

The general rule for many years was that a prodrug of a controlled substance was placed in the same CSA schedule as its active metabolite, for example, lisdexamfetamine and d-amphetamine are both in C-II. There have been exceptions, for example, propofol is not scheduled, but its prodrug, fospropofol, is in C-IV, and d-*threo*-methylphenidate is in C-II, but its prodrug, serdexmethylphenidate, is in C-IV. Within the “classic” psychedelics class, both psilocybin (prodrug) and psilocin (active metabolite) are in C-I. If other psychedelic prodrugs emerge in clinical development, they will be treated as “novel” psychedelics and their risk of abuse and dependence will be benchmarked against its active metabolite.

The sponsor’s proposal and FDA’s determination of CSA scheduling is made according to an integrated assessment of the abuse and dependence risks of the drug-candidate across these eight factors. The procedures in other countries employ variations on this procedure but the fundamentals remain the same.

## Scheduling under the U.S. CSA

Although Johnson et al. (2018) proposed that the “classic” psychedelic, psilocybin, was compatible with C-IV based on an 8-Factor Analysis assessment, any evaluation of a psychedelic drug-candidate is outside of the scope of this review. Our view is the first psychedelic, which is likely to be a member of the “classic” psychedelics class, will set the precedent and all conventional psychedelic drugs that follow are very likely to be assigned to the same CSA schedule. The experience gained from the benzodiazepines and GABA-A receptor PAMs predicts that novel psychedelic drug-candidates will have to demonstrate substantial differences in the risk of abuse/dependence to achieve an alternative scheduling outcome.

Psychedelic compounds that are not currently included in C-I, for example, 5MeO-DMT and DOI, will almost certainly lose their non-scheduled status if they are approved for medical use and will likely be placed in the same schedule as other medically-approved “classic” and “novel” psychedelic drugs.

Although the decision on controlled drug scheduling is based on an objective and dispassionate evaluation of the non-clinical, clinical, and epidemiological evidence of the abuse and dependence risks related to the drug-candidate, there will inevitably be a political dimension to the scheduling decision; this will especially be the case given the extensive human use of the “classic” psychedelics. There is likely to be considerable variability across countries depending on their individual attitude to the abuse potential risk deriving from their adoption as drugs for human use. That said, our prediction is that the degree of controlled drug restriction that is assigned to the first psychedelic drugs to be approved will apply to all subsequent psychedelic drugs that have typical 5-HT_2A_ receptor agonist properties.

## Conclusions

Pharmaceutical research in the psychedelics space is moving at great speed, and as it progresses, it creates new challenges for drug developers, regulatory authorities, legislators, prescribers, patients, and the public. Several of the “classic” psychedelics which initiated research into the therapeutic potential of this drug class are now in Phase 3 clinical trials with a view to regulatory approval within the next few years. In the search for improvements, innovation, and intellectual property, the field is transitioning to the next generation of molecules, and this change is taking place before the first psychedelic drug has received formal approval for medical use in humans. Given that the United States is the most important market for many drug developers, FDA is likely to undertake the first regulatory assessment of a “classic” psychedelic drug-candidate. Given its pivotal role, FDA has led the field in disseminating advice on how to address the clinical and regulatory challenges posed by the psychedelics (Calderon et al., 2018, 2023; CDER/FDA, 2023). In this review, we have built on this foundation by covering areas that are unclear or have not been discussed and offer what we hope will be helpful and constructive proposals to address some of these points. We have also set out a framework for classifying novel psychedelics so their risks for abuse can be systematically investigated, benchmarked, and reflected in appropriate controlled drug scheduling.

## References

[bibr1-02698811251382147] Abramson HA Jarvik ME Gorin MH , et al. (1956) Lysergic acid diethylamide (LSD-25): XVII. Tolerance development and its relationship to a theory of psychosis. The Journal of Psychology 41(1): 81–105.

[bibr2-02698811251382147] Anglin D Spears KL Hutson HR (1997) Flunitrazepam and its involvement in date or acquaintance rape. Academic Emergency Medicine: Official Journal of the Society for Academic Emergency Medicine 4(4): 323–326. 9107334 10.1111/j.1553-2712.1997.tb03557.x

[bibr3-02698811251382147] Arnt J (1989) Characterization of the discriminative stimulus properties induced by 5-HT1 and 5-HT2 agonists in rats. Pharmacology & Toxicology 64(2): 165–172. 2526950 10.1111/j.1600-0773.1989.tb00623.x

[bibr4-02698811251382147] Arnt J Hyttel J (1989) Facilitation of 8-OHDPAT-induced forepaw treading of rats by the 5-HT2 agonist DOI. European Journal of Pharmacology 161(1): 45–51. 2524390 10.1016/0014-2999(89)90178-7

[bibr5-02698811251382147] Bedard P Pycock CJ (1977) “Wet-dog” shake behaviour in the rat: A possible quantitative model of central 5-hydroxytryptamine activity. Neuropharmacology 16(10): 663–670. 304190 10.1016/0028-3908(77)90117-4

[bibr6-02698811251382147] Bergman U Griffiths RR (1986) Relative abuse of diazepam and oxazepam: Prescription forgeries and theft/loss reports in Sweden. Drug and Alcohol Dependence 16(4): 293–301. 3486104 10.1016/0376-8716(86)90063-3

[bibr7-02698811251382147] Bistue Millón MB Noguera L Bruno D , et al. (2025) Safety and tolerability of multiple sublingual microdoses of 5-MeO-DMT in adults with moderate symptoms of depression and/or anxiety: A randomized, double-blind, placebo-controlled study. Neuropsychopharmacol 50: 1715–1723. 10.1038/s41386-025-02167-3 PMC12436647 40659913

[bibr8-02698811251382147] Bonson KR (2018) Regulation of human research with LSD in the United States (1949–1987). Psychopharmacology 235(2): 591–604. 29147729 10.1007/s00213-017-4777-4

[bibr9-02698811251382147] Bressloff PC Cowan JD Golubitsky M , et al. (2002) What geometric visual hallucinations tell us about the visual cortex. Neural Computation 14(3): 473–491. 11860679 10.1162/089976602317250861

[bibr10-02698811251382147] Calderon SN Bonson KR Reissig CJ , et al. (2023) Considerations in assessing the abuse potential of psychedelics during drug development. Neuropharmacology 224: 109352. 36455646 10.1016/j.neuropharm.2022.109352

[bibr11-02698811251382147] Calderon SN Giarola A Heal DJ (2015) Regulatory framework and guidance to the evaluation of the abuse liability of drugs in the United States and Europe. In: Markgraf CG Hudzik TJ Compton DR (eds) Nonclinical Assessment of Abuse Potential for New Pharmaceuticals. Academic Press, pp.245–268.

[bibr12-02698811251382147] Calderon SN Hunt J Klein M (2018) A regulatory perspective on the evaluation of hallucinogen drugs for human use. Neuropharmacology 142: 135–142. 29180224 10.1016/j.neuropharm.2017.11.028

[bibr13-02698811251382147] Calderon SN , et al. (2020) Evaluation of HAP studies for drugs with novel mechanisms of action. In: Poster presented at The College on Problems of Drug Dependence (CPDD) annual meeting 2020 (virtual).

[bibr14-02698811251382147] Calhoun SR Wesson DR Galloway GP , et al. (1996) Abuse of flunitrazepam (Rohypnol) and other benzodiazepines in Austin and south Texas. Journal of Psychoactive Drugs 28(2): 183–189. 8811586 10.1080/02791072.1996.10524390

[bibr15-02698811251382147] Cardona-Acosta AM Meisser N Vardeleon NI , et al. (2025) Mother’s little helper turned a foe: Alprazolam use, misuse, and abuse. Progress in Neuro-Psychopharmacology & Biological Psychiatry 136: 111137. 39260815 10.1016/j.pnpbp.2024.111137

[bibr16-02698811251382147] Caro Y Calderon S Chen L , et al. (2022) Human abuse potential study results in the context of abuse detected postmarketing. In: Poster presented at The College on Problems of Drug Dependence (CPDD) annual meeting 2022, Minneapolis, MN, 11–15 June.

[bibr17-02698811251382147] CDER/FDA (2017) U.S. Department of Health and Human Services, Food and Drug Administration, Centre for Drug Evaluation and Research. Assessment of abuse potential of drugs. Guidance for industry. Available at: www.fda.gov/downloads/drugs/guidancecomplianceregulatoryinformation/guidances/ucm334743.pdf (accessed 24 April 2025).

[bibr18-02698811251382147] CDER/FDA (2023) U.S. Department of Health and Human Services, Food and Drug Administration, Centre for Drug Evaluation and Research. Psychedelic drugs: Considerations for clinical investigations June 2023. Available at: https://www.fda.gov/regulatory-information/search-fda-guidance-documents/psychedelic-drugs-considerations-clinical-investigations (accessed 24 April 2025).

[bibr19-02698811251382147] CHMP/EMA (2006) European Medicine Agency, Committee for Medicinal Products for Human Use. Guidance of the non-clinical investigation of the dependence potential of medicinal products. Available at: http://www.ema.europa.eu/docs/en_GB/document_library/Scientific_guideline/2009/09/WC500003360.pdf (accessed 24 April 2025).

[bibr20-02698811251382147] Chojnacka-Wójcik E Kłodzińska A (1997) Involvement of 5-hydroxytryptamine 2A (5-HT2A) receptors in the mediation of the discriminative stimulus properties of (+/−)DOI in rats. Polish Journal of Pharmacology 49(5): 299–304. 9566028

[bibr21-02698811251382147] Cholden LS Kurland A Savage C (1955) Clinical reactions and tolerance to LSD in chronic schizophrenia. The Journal of Nervous and Mental Disease 122(3): 211–221. 13295823 10.1097/00005053-195509000-00001

[bibr22-02698811251382147] Colpaert FC Niemegeers CJ Janssen PA (1982) A drug discrimination analysis of lysergic acid diethylamide (LSD): In vivo agonist and antagonist effects of purported 5-hydroxytryptamine antagonists and of pirenperone, a LSD-antagonist. The Journal of Pharmacology and Experimental Therapeutics 221(1): 206–214. 7062283

[bibr23-02698811251382147] Contreras A Khumnark M Hines RM , et al. (2021) Behavioral arrest and a characteristic slow waveform are hallmark responses to selective 5-HT_2A_ receptor activation. Scientific Reports 11(1): 1925. 33479368 10.1038/s41598-021-81552-6 PMC7820508

[bibr24-02698811251382147] Corne SJ Pickering RW (1967) A possible correlation between drug-induced hallucinations in man and a behavioural response in mice. Psychopharmacologia 11(1): 65–78. 5302272 10.1007/BF00401509

[bibr25-02698811251382147] Corne SJ Pickering RW Warner BT (1963) A method for assessing the effects of drugs on the central actions of 5-hydroxytryptamine. British Journal of Pharmacology and Chemotherapy 20(1): 106–120. 14023050 10.1111/j.1476-5381.1963.tb01302.x PMC1703746

[bibr26-02698811251382147] Creehan KM Vandewater SA Taffe MA (2015) Intravenous self-administration of mephedrone, methylone and MDMA in female rats. Neuropharmacology 92: 90–97. 25600245 10.1016/j.neuropharm.2015.01.003 PMC4346510

[bibr27-02698811251382147] Cunningham KA Appel JB (1987) Neuropharmacological reassessment of the discriminative stimulus properties of d-lysergic acid diethylamide (LSD). Psychopharmacology 91(1): 67–73. 3103161 10.1007/BF00690929

[bibr28-02698811251382147] Custodio RJP Sayson LV Botanas CJ , et al. (2020) 25B-NBOMe, a novel N-2-methoxybenzyl-phenethylamine (NBOMe) derivative, may induce rewarding and reinforcing effects via a dopaminergic mechanism: Evidence of abuse potential. Addiction Biology 25(6): e12850. 10.1111/adb.12850 31749223

[bibr29-02698811251382147] Darmani NA Martin BR Glennon RA (1992) Behavioral evidence for differential adaptation of the serotonergic system after acute and chronic treatment with (+/−)-1-(2,5-dimethoxy-4-iodophenyl)-2-aminopropane (DOI) or ketanserin. The Journal of Pharmacology and Experimental Therapeutics 262(2): 692–698. 1501117

[bibr30-02698811251382147] Davis AK Barsuglia JP Lancelotta R , et al. (2018) The epidemiology of 5-methoxy-N, N-dimethyltryptamine (5-MeO-DMT) use: Benefits, consequences, patterns of use, subjective effects, and reasons for consumption. Journal of Psychopharmacology 32(7): 779–792. 29708042 10.1177/0269881118769063 PMC6248886

[bibr31-02698811251382147] Dowd SM Strong MJ Janicak PG , et al. (2002) The behavioral and cognitive effects of two benzodiazepines associated with drug-facilitated sexual assault. Journal of Forensic Sciences 47(5): 1101–1107. 12353555

[bibr32-02698811251382147] Dourron HM Nichols CD Simonsson O , et al. (2023) 5-MeO-DMT: An atypical psychedelic with unique pharmacology, phenomenology & risk? Psychopharmacology 242: 1457–1479. 38072874 10.1007/s00213-023-06517-1

[bibr33-02698811251382147] Ermakova AO Dunbar F Rucker J , et al. (2022) A narrative synthesis of research with 5-MeO-DMT. Journal of Psychopharmacology (Oxford, England) 36(3): 273–294. 34666554 10.1177/02698811211050543 PMC8902691

[bibr34-02698811251382147] Erowid (n.d.a) 5-MeO-DMT effects. Available at: https://erowid.org/chemicals/5meo_dmt/5meo_dmt_effects.shtml (accessed 24 April 2025).

[bibr35-02698811251382147] Erowid (n.d.b) DMT effects. Available at: https://erowid.org/chemicals/dmt/dmt_effects.shtml (accessed 24 April 2025).

[bibr36-02698811251382147] Eshleman AJ Forster MJ Wolfrum KM , et al. (2014) Behavioral and neurochemical pharmacology of six psychoactive substituted phenethylamines: Mouse locomotion, rat drug discrimination and in vitro receptor and transporter binding and function. Psychopharmacology 231(5): 875–888. 24142203 10.1007/s00213-013-3303-6 PMC3945162

[bibr37-02698811251382147] Fantegrossi WE Woods JH Winger G (2004) Transient reinforcing effects of phenylisopropylamine and indolealkylamine hallucinogens in rhesus monkeys. Behavioural Pharmacology 15(2): 149–157. 15096915 10.1097/00008877-200403000-00007

[bibr38-02698811251382147] FDA (2020) FDA requiring Boxed Warning updated to improve safe use of benzodiazepine drug class. Available at: https://www.fda.gov/drugs/drug-safety-and-availability/fda-requiring-boxed-warning-updated-improve-safe-use-benzodiazepine-drug-class (accessed 30 April 2025).

[bibr39-02698811251382147] Forrester MB (2006) Flunitrazepam abuse and malicious use in Texas, 1998–2003. Substance Use & Misuse 41(3): 297–306. 16467007 10.1080/10826080500409134

[bibr40-02698811251382147] Galati S (2023) Identifying relevant adverse events of interest and recommendations for analysis and presentation of data in the NDA submission. In: Presentation at the Cross-Company Abuse Liability Council (C-CALC) meeting “Advancements and challenges in abuse potential evaluation 2023,” September 2023, pp.27–28. Hilton Hotel and Executive Meeting Center. Available at: https://img1.wsimg.com/blobby/go/933911af-c640-4678-bef3-07ff76ae9c47/downloads/Topic%203%20%20AEs%20and%20Analysis_Steven%20Galati%20FDA.pdf?ver=1705683910630 (accessed 24 April 2025).

[bibr41-02698811251382147] Garel N Tate S Nash K , et al. (2024) Trends in hallucinogen-associated emergency department visits and hospitalizations in California, USA, from 2016 to 2022. Addiction 119(5): 960–964. 38213013 10.1111/add.16432

[bibr42-02698811251382147] Gatch MB Kozlenkov A Huang RQ , et al. (2013) The HIV antiretroviral drug efavirenz has LSD-like properties. Neuropsychopharmacology: Official Publication of the American College of Neuropsychopharmacology 38(12): 2373–2384. 23702798 10.1038/npp.2013.135 PMC3799056

[bibr43-02698811251382147] Gautam L Sharratt SD Cole MD (2014) Drug facilitated sexual assault: Detection and stability of benzodiazepines in spiked drinks using gas chromatography-mass spectrometry. PLoS One 9(2): e89031. 10.1371/journal.pone.0089031 PMC3929633 24586489

[bibr44-02698811251382147] Gauvin DV Baird TJ (2008) A functional observational battery in non-human primates for regulatory-required neurobehavioral assessments. Journal of Pharmacological and Toxicological Methods 58(2): 88–93. 18586529 10.1016/j.vascn.2008.05.002

[bibr45-02698811251382147] Glennon RA Liebowitz SM Leming-Doot D , et al. (1980) Demethyl analogues of psychoactive methoxyphenalkylamines: Synthesis and serotonin receptor affinities. Journal of Medicinal Chemistry 23(9): 990–994. 7411554 10.1021/jm00183a006

[bibr46-02698811251382147] Glennon RA Rosecrans JA Young R , et al. (1979) Hallucinogens as a discriminative stimuli: Generalization of DOM to a 5-methoxy-N, N-dimethyltryptamine stimulus. Life Sciences 24(11): 993–997. 286864 10.1016/0024-3205(79)90317-5

[bibr47-02698811251382147] Glennon RA Titeler M McKenney JD . (1984) Evidence for 5-HT_2_ involvement in the mechanism of action of hallucinogenic agents. Life Sciences 35: 2502–2511. 10.1016/0024-3205(84)90436-3 6513725

[bibr48-02698811251382147] Glennon RA Young R Jacyno JM , et al. (1983) DOM-stimulus generalization to LSD and other hallucinogenic indolealkylamines. European Journal of Pharmacology 86(3–4): 453–459. 6572591 10.1016/0014-2999(83)90196-6

[bibr49-02698811251382147] González-Maeso J Weisstaub NV Zhou M , et al. (2007) Hallucinogens recruit specific cortical 5-HT(2A) receptor-mediated signaling pathways to affect behavior. Neuron 53(3): 439–452. 17270739 10.1016/j.neuron.2007.01.008

[bibr50-02698811251382147] Goodwin AK (2016) An intravenous self-administration procedure for assessing the reinforcing effects of hallucinogens in nonhuman primates. Journal of Pharmacological and Toxicological Methods 82: 31–36. 27473331 10.1016/j.vascn.2016.07.004

[bibr51-02698811251382147] Goodwin AK Pynnonen DM Baker LE (2003) Serotonergic-dopaminergic mediation of MDMA’s discriminative stimulus effects in a three-choice discrimination. Pharmacology, Biochemistry, and Behavior 74(4): 987–995. 12667914 10.1016/s0091-3057(03)00029-7

[bibr52-02698811251382147] Goodwin GM Aaronson ST Alvarez O , et al. (2022) Single-dose psilocybin for a treatment-resistant episode of major depression. The New England Journal of Medicine 387(18): 1637–1648. 36322843 10.1056/NEJMoa2206443

[bibr53-02698811251382147] Gouzoulis-Mayfrank E Heekeren K Neukirch A , et al. (2005) Psychological effects of (S)-ketamine and N,N-dimethyltryptamine (DMT): A double-blind, cross-over study in healthy volunteers. Pharmacopsychiatry 38(6): 301–311. 16342002 10.1055/s-2005-916185

[bibr54-02698811251382147] Green AR Heal DJ (1985) The effect of drugs on serotonin-mediated behavioural models. In: Green AR (ed.) Neuropharmacology of Serotonin. Oxford University Press, pp.326–365.

[bibr55-02698811251382147] Gresch PJ Barrett RJ Sanders-Bush E , et al. (2007) 5-Hydroxytryptamine (serotonin)2A receptors in rat anterior cingulate cortex mediate the discriminative stimulus properties of d-lysergic acid diethylamide. Journal of Pharmacology and Experimental Therapeutics 320(2): 662–669. 17077317 10.1124/jpet.106.112946

[bibr56-02698811251382147] Griffiths RR McLeod DR Bigelow GE , et al. (1984) Comparison of diazepam and oxazepam: Preference, liking and extent of abuse. The Journal of Pharmacology and Experimental Therapeutics 229(2): 501–508. 6716272

[bibr57-02698811251382147] Griffiths RR Wolf B (1990) Relative abuse liability of different benzodiazepines in drug abusers. Journal of Clinical Psychopharmacology 10(4): 237–243. 1981067

[bibr58-02698811251382147] Halberstadt AL Geyer MA (2014) Effects of the hallucinogen 2,5-dimethoxy-4-iodophenethylamine (2C-I) and superpotent N-benzyl derivatives on the head twitch response. Neuropharmacology 77: 200–207. 24012658 10.1016/j.neuropharm.2013.08.025 PMC3866097

[bibr59-02698811251382147] Halberstadt AL Chatha M Klein AK , et al. (2020) Correlation between the potency of hallucinogens in the mouse head-twitch response assay and their behavioral and subjective effects in other species. Neuropharmacology 167: 107933. 31917152 10.1016/j.neuropharm.2019.107933 PMC9191653

[bibr60-02698811251382147] Halberstadt AL Nichols DE Geyer MA (2012) Behavioral effects of α,α,β,β-tetradeutero-5-MeO-DMT in rats: Comparison with 5-MeO-DMT administered in combination with a monoamine oxidase inhibitor. Psychopharmacology 221(4): 709–718. 22222861 10.1007/s00213-011-2616-6 PMC3796951

[bibr61-02698811251382147] Heal DJ Gosden J Smith SL (2018) Evaluating the abuse potential of psychedelic drugs as part of the safety pharmacology assessment for medical use in humans. Neuropharmacology 142: 89–115. 29427652 10.1016/j.neuropharm.2018.01.049

[bibr62-02698811251382147] Heal DJ Gosden J Smith SL , et al. (2023) Experimental strategies to discover and develop the next generation of psychedelics and entactogens as medicines. Neuropharmacology 225: 109375. 36529260 10.1016/j.neuropharm.2022.109375

[bibr63-02698811251382147] Heal DJ Luscombe GP Martin KF (1992) Pharmacological identification of 5-HT receptor subtypes using behavioural models. In: Marsden CA Heal DJ (eds) Central Serotonin Receptors and Psychotropic Drugs. Blackwell Scientific, pp.56–99.

[bibr64-02698811251382147] Heal DJ Philpot J Molyneux SG , et al. (1985) Intracerebroventricular administration of 5,7-dihydroxytryptamine to mice increases both head-twitch response and the number of cortical 5-HT2 receptors. Neuropharmacology 24(12): 1201–1205. 4094656 10.1016/0028-3908(85)90155-8

[bibr65-02698811251382147] Heal DJ Philpot J O’Shaughnessy KM , et al. (1986) The influence of central noradrenergic function on 5-HT2-mediated head-twitch responses in mice: Possible implications for the actions of antidepressant drugs. Psychopharmacology 89(4): 414–420. 3018823 10.1007/BF02412113

[bibr66-02698811251382147] Heal DJ Smith SL Gosden J , et al. (2025) Discriminating evidence – Use and misuse of the drug-discrimination test in abuse potential assessment of novel CNS drugs. Journal of Psychopharmacology (Oxford, England) 39: 629–651. 40243002 10.1177/02698811251330780 PMC12267871

[bibr67-02698811251382147] Henningfield JE Ashworth J Heal DJ , et al. (2023) Psychedelic drug abuse potential assessment for new drug applications and controlled substance scheduling: A United States perspective. Journal of Psychopharmacology (Oxford, England) 37(1): 33–44. 36588452 10.1177/02698811221140004

[bibr68-02698811251382147] Henningfield JE Coe MA Griffiths RR , et al. (2022) Psychedelic drug abuse potential assessment research for new drug applications and Controlled Substances Act scheduling. Neuropharmacology 218: 109220. 35987353 10.1016/j.neuropharm.2022.109220

[bibr69-02698811251382147] Holze F Singh N Liechti ME , et al. (2024) Serotonergic psychedelics: A comparative review of efficacy, safety, pharmacokinetics, and binding profile. Biological Psychiatry: Cognitive Neuroscience and Neuroimaging 9(5): 472–489. 38301886 10.1016/j.bpsc.2024.01.007

[bibr70-02698811251382147] Holze F Vizeli P Müller F , et al. (2020) Distinct acute effects of LSD, MDMA, and D-amphetamine in healthy subjects. Neuropsychopharmacology: Official Publication of the American College of Neuropsychopharmacology 45(3): 462–471. 31733631 10.1038/s41386-019-0569-3 PMC6969135

[bibr71-02698811251382147] Honk L Stenfors CUD Goldberg SB , et al. (2024) Longitudinal associations between psychedelic use and psychotic symptoms in the United States and the United Kingdom. Journal of Affective Disorders 351, 194–201. 38280572 10.1016/j.jad.2024.01.197 PMC10922895

[bibr72-02698811251382147] Isbell H Miner EJ Logan CR (1959) Cross tolerance between D-2-brom-lysergic acid diethylamide (BOL-148) and the D-diethylamide of lysergic acid (LSD-25). Psychopharmacologia 1: 109–116. 14405871 10.1007/BF00409110

[bibr73-02698811251382147] Ismaiel AM De Los Angeles J Teitler M , et al. (1993) Antagonism of 1-(2,5-dimethoxy-4-methylphenyl)-2-aminopropane stimulus with a newly identified 5-HT2-versus 5-HT1C-selective antagonist. Journal of Medicinal Chemistry 36(17): 2519–2525. 8355253 10.1021/jm00069a010

[bibr74-02698811251382147] Jennings KA Sheward WJ Harmar AJ , et al. (2008) Evidence that genetic variation in 5-HT transporter expression is linked to changes in 5-HT2A receptor function. Neuropharmacology 54(5): 776–783. 18241894 10.1016/j.neuropharm.2007.12.001

[bibr75-02698811251382147] Johnson MW Griffiths RR Hendricks PS , et al. (2018) The abuse potential of medical psilocybin according to the 8 factors of the Controlled Substances Act. Neuropharmacology 142: 143–166. 29753748 10.1016/j.neuropharm.2018.05.012 PMC6791528

[bibr76-02698811251382147] Johnson MW Richards W Griffiths R (2008) Human hallucinogen research: Guidelines for safety. Journal of Psychopharmacology 22(6): 603–620. 18593734 10.1177/0269881108093587 PMC3056407

[bibr77-02698811251382147] Kangaslampi S (2023) Association between mystical-type experiences under psychedelics and improvements in well-being or mental health – A comprehensive review of the evidence. Journal of Psychedelic Studies 7: 18–28.

[bibr78-02698811251382147] Kintz P Villain M Dumestre-Toulet V , et al. (2005) Drug-facilitated sexual assault and analytical toxicology: The role of LC-MS/MS A case involving zolpidem. Journal of Clinical Forensic Medicine 12(1): 36–41. 15763689 10.1016/j.jcfm.2004.08.005

[bibr79-02698811251382147] Ko K Knight G Rucker JJ , et al. (2022) Psychedelics, mystical experience, and therapeutic efficacy: A systematic review. Frontiers in Psychiatry 13: 917199. 35923458 10.3389/fpsyt.2022.917199 PMC9340494

[bibr80-02698811251382147] Kopra EI Penttinen J Rucker JJ , et al. (2025) Psychedelic-related deaths in England, Wales and Northern Ireland (1997–2022). Progress in Neuro-Psychopharmacology & Biological Psychiatry 136: 111177. 39437962 10.1016/j.pnpbp.2024.111177

[bibr81-02698811251382147] Kueppers VB Cooke CT (2015) 25I-NBOMe related death in Australia: A case report. Forensic Science International 249: e15–e18. 10.1016/j.forsciint.2015.02.010 25747271

[bibr82-02698811251382147] Le GH Wong S Haikazian S , et al. (2024) Association between cognitive functioning, suicidal ideation and suicide attempts in major depressive disorder, bipolar disorder, schizophrenia and related disorders: A systematic review and meta-analysis. Journal of Affective Disorders 365: 381–399. 39168166 10.1016/j.jad.2024.08.057

[bibr83-02698811251382147] Lebedev AV Acar K Garzón B , et al. (2021) Psychedelic drug use and schizotypy in young adults. Scientific Reports 11(1): 15058. 34301969 10.1038/s41598-021-94421-z PMC8302700

[bibr84-02698811251382147] Lerner M Lyvers M (2006) Values and beliefs of psychedelic drug users: A cross-cultural study. Journal of Psychoactive Drugs 38(2): 143–147. 16903453 10.1080/02791072.2006.10399838

[bibr85-02698811251382147] Leysen JE Janssen PF Niemegeers CJ (1989) Rapid desensitization and down-regulation of 5-HT2 receptors by DOM treatment. European Journal of Pharmacology 163(1): 145–149. 2545460 10.1016/0014-2999(89)90409-3

[bibr86-02698811251382147] Lintzeris N Mitchell TB Bond AJ , et al. (2007) Pharmacodynamics of diazepam co-administered with methadone or buprenorphine under high dose conditions in opioid dependent patients. Drug and Alcohol Dependence 91(2–3): 187–194. 17624687 10.1016/j.drugalcdep.2007.05.019

[bibr87-02698811251382147] Malcolm B Thomas K (2022) Serotonin toxicity of serotonergic psychedelics. Psychopharmacology 239(6): 1881–1891. 34251464 10.1007/s00213-021-05876-x

[bibr88-02698811251382147] Marona-Lewicka D Chemel BR Nichols DE (2009) Dopamine D4 receptor involvement in the discriminative stimulus effects in rats of LSD, but not the phenethylamine hallucinogen DOI. Psychopharmacology 203(2): 265–277. 18604600 10.1007/s00213-008-1238-0

[bibr89-02698811251382147] Mateu-Gelabert P Jessell L Goodbody E , et al. (2017) High enhancer, downer, withdrawal helper: Multifunctional nonmedical benzodiazepine use among young adult opioid users in New York City. The International Journal on Drug Policy 46: 17–27. 28577506 10.1016/j.drugpo.2017.05.016 PMC5609816

[bibr90-02698811251382147] Meshkat S Malik T Zeifman R , et al. (2025) Psychedelics and suicide-related outcomes: A systematic review. Journal of Clinical Medicine 14(5): 1416. 40094838 10.3390/jcm14051416 PMC11900607

[bibr91-02698811251382147] Metz A Heal DJ (1986) In mice repeated administration of electroconvulsive shock or desmethylimipramine produces rapid alterations in 5-HT2-mediated head-twitch responses and cortical 5-HT2 receptor number. European Journal of Pharmacology 126(1–2): 159–162. 3758161 10.1016/0014-2999(86)90754-5

[bibr92-02698811251382147] Millière R Carhart-Harris RL Roseman L , et al. (2018) Psychedelics, meditation, and self-consciousness. Frontiers in Psychology 9: 1475. 30245648 10.3389/fpsyg.2018.01475 PMC6137697

[bibr93-02698811251382147] Moitra M Santomauro D Degenhardt L , et al. (2021) Estimating the risk of suicide associated with mental disorders: A systematic review and meta-regression analysis. Journal of Psychiatric Research 137: 242–249. 33714076 10.1016/j.jpsychires.2021.02.053 PMC8095367

[bibr94-02698811251382147] Mortaheb S Fort LD Mason NL , et al. (2024) Dynamic functional hyperconnectivity after psilocybin intake is primarily associated with oceanic boundlessness. Biological Psychiatry: Cognitive Neuroscience and Neuroimaging 9(7): 681–692. 38588855 10.1016/j.bpsc.2024.04.001

[bibr95-02698811251382147] Myran DT Pugliese M Xiao J , et al. (2025) Emergency department visits involving hallucinogen use and risk of schizophrenia spectrum disorder. JAMA Psychiatry 82(2): 142–150. 39535804 10.1001/jamapsychiatry.2024.3532 PMC11561722

[bibr96-02698811251382147] Olson DE (2020) The subjective effects of psychedelics may not be necessary for their enduring therapeutic effects. ACS Pharmacology & Translational Science 4(2): 563–567. 33861218 10.1021/acsptsci.0c00192 PMC8033607

[bibr97-02698811251382147] Pérez Orts M van Asten A Kohler I (2023) The evolution toward designer benzodiazepines in drug-facilitated sexual assault cases. Journal of Analytical Toxicology 47(1): 1–25. 35294022 10.1093/jat/bkac017 PMC9942444

[bibr98-02698811251382147] Preller KH Burt JB Ji JL , et al. (2018) Changes in global and thalamic brain connectivity in LSD-induced altered states of consciousness are attributable to the 5-HT2A receptor. Elife 7: e35082. 10.7554/eLife.35082 PMC6202055 30355445

[bibr99-02698811251382147] Preller KH Herdener M Pokorny T , et al. (2017) The fabric of meaning and subjective effects in LSD-induced states depend on serotonin 2A receptor activation. Current Biology 27: 451–457. 28132813 10.1016/j.cub.2016.12.030

[bibr100-02698811251382147] Preller KH Razi A Zeidman P , et al. (2019) Effective connectivity changes in LSD-induced altered states of consciousness in humans. Proceedings of the National Academy of Sciences of the United States of America 116: 2743–2748. 30692255 10.1073/pnas.1815129116 PMC6377471

[bibr101-02698811251382147] Preston KL Griffiths RR Stitzer ML , et al. (1984) Diazepam and methadone interactions in methadone maintenance. Clinical Pharmacology and Therapeutics 36(4): 534–541. 6478738 10.1038/clpt.1984.215

[bibr102-02698811251382147] Ray TS (2010) Psychedelics and the human receptorome. PLoS One 5(2): e9019. 10.1371/journal.pone.0009019 PMC2814854 20126400

[bibr103-02698811251382147] Reckweg JT Uthaug MV Szabo A , et al. (2022) The clinical pharmacology and potential therapeutic applications of 5-methoxy-N,N-dimethyltryptamine (5-MeO-DMT). Journal of Neurochemistry 162(1): 128–146. 35149998 10.1111/jnc.15587 PMC9314805

[bibr104-02698811251382147] Reckweg JT van Leeuwen CJ Henquet C , et al. (2023) A phase 1/2 trial to assess safety and efficacy of a vaporized 5-methoxy-N,N-dimethyltryptamine formulation (GH001) in patients with treatment-resistant depression. Frontiers in Psychiatry 14: 1133414. 37409159 10.3389/fpsyt.2023.1133414 PMC10319409

[bibr105-02698811251382147] Rickli A Moning OD Hoener MC , et al. (2016) Receptor interaction profiles of novel psychoactive tryptamines compared with classic hallucinogens. European Neuropsychopharmacology 26(8): 1327–1337. 27216487 10.1016/j.euroneuro.2016.05.001

[bibr106-02698811251382147] Romeo B Kervadec E Fauvel B , et al. (2025) Exploring factors associated with the intensity of a mystical experience following naturalistic psychedelic use: A retrospective survey. Progress in Neuro-Psychopharmacology & Biological Psychiatry 137: 111300. 40010428 10.1016/j.pnpbp.2025.111300

[bibr107-02698811251382147] Rose SR Poklis JL Poklis A (2013) A case of 25I-NBOMe (25-I) intoxication: A new potent 5-HT2A agonist designer drug. Clinical Toxicology (Philadelphia, PA) 51(3): 174–177. 23473462 10.3109/15563650.2013.772191 PMC4002208

[bibr108-02698811251382147] Rothman RB Baumann MH Savage JE , et al. (2000) Evidence for possible involvement of 5-HT(2B) receptors in the cardiac valvulopathy associated with fenfluramine and other serotonergic medications. Circulation 102(23): 2836–2841. 11104741 10.1161/01.cir.102.23.2836

[bibr109-02698811251382147] Rucker JJ Roberts C Seynaeve M , et al. (2024) Phase 1, placebo-controlled, single ascending dose trial to evaluate the safety, pharmacokinetics and effect on altered states of consciousness of intranasal BPL-003 (5-methoxy-*N,N*-dimethyltryptamine benzoate) in healthy participants. Journal of Psychopharmacology (Oxford, England) 38(8): 712–723. 38616411 10.1177/02698811241246857 PMC11311898

[bibr110-02698811251382147] Salomone A Gerace E Di Corcia D , et al. (2012) Hair analysis of drugs involved in drug-facilitated sexual assault and detection of zolpidem in a suspected case. International Journal of Legal Medicine 126(3): 451–459. 21751027 10.1007/s00414-011-0597-y

[bibr111-02698811251382147] Schmitz A (2016) Benzodiazepine use, misuse, and abuse: A review. The Mental Health Clinician 6(3): 120–126. 29955458 10.9740/mhc.2016.05.120 PMC6007645

[bibr112-02698811251382147] Schindler EA Dave KD Smolock EM , et al. (2012) Serotonergic and dopaminergic distinctions in the behavioral pharmacology of (±)-1-(2,5-dimethoxy-4-iodophenyl)-2-aminopropane (DOI) and lysergic acid diethylamide (LSD) Pharmacology, Biochemistry, and Behavior 101(1): 69–76. 22197710 10.1016/j.pbb.2011.12.002 PMC3272148

[bibr113-02698811251382147] Schreiber R de Vry J (1993) Studies on the neuronal circuits involved in the discriminative stimulus effects of 5-hydroxytryptamine1A receptor agonists in the rat. The Journal of Pharmacology and Experimental Therapeutics 265(2): 572–579. 8496807

[bibr114-02698811251382147] Schreiber R Brocco M Millan MJ (1994) Blockade of the discriminative stimulus effects of DOI by MDL 100,907 and the “atypical” antipsychotics, clozapine and risperidone. European Journal of Pharmacology 264(1): 99–102. 7530204 10.1016/0014-2999(94)90643-2

[bibr115-02698811251382147] Setola V Dukat M Glennon RA , et al. (2005) Molecular determinants for the interaction of the valvulopathic anorexigen norfenfluramine with the 5-HT2B receptor. Molecular Pharmacology 68(1): 20–33. 15831837 10.1124/mol.104.009266

[bibr116-02698811251382147] Schwartz RH Milteer R LeBeau MA (2000) Drug-facilitated sexual assault (“date rape”). Southern Medical Journal 93(6): 558–561. 10881768

[bibr117-02698811251382147] Smith RL Barrett RJ Sanders-Bush E (2003) Discriminative stimulus properties of 1-(2,5-dimethoxy-4-iodophenyl)-2-aminopropane [(+/−)DOI] in C57BL/6J mice. Psychopharmacology 166(1): 61–68. 12474110 10.1007/s00213-002-1252-6

[bibr118-02698811251382147] Spencer DG Jr Glaser T Traber J (1987) Serotonin receptor subtype mediation of the interoceptive discriminative stimuli induced by 5-methoxy-N,N-dimethyltryptamine. Psychopharmacology 93(2): 158–166. 3122248 10.1007/BF00179927

[bibr119-02698811251382147] Sutar R Kumar A Yadav V (2023) Suicide and prevalence of mental disorders: A systematic review and meta-analysis of world data on case-control psychological autopsy studies. Psychiatry Research 329: 115492. 37783094 10.1016/j.psychres.2023.115492

[bibr120-02698811251382147] Titeler M Lyon RA Glennon RA (1988) Radioligand binding evidence implicates the brain 5-HT2 receptor as a site of action for LSD and phenylisopropylamine hallucinogens. Psychopharmacology 94: 213–216. 3127847 10.1007/BF00176847

[bibr121-02698811251382147] Too LS Spittal MJ Bugeja L , et al. (2019) The association between mental disorders and suicide: A systematic review and meta-analysis of record linkage studies. Journal of Affective Disorders 259: 302–313. 31450139 10.1016/j.jad.2019.08.054

[bibr122-02698811251382147] United Nations (1971) Convention on psychotropic substances treaty. Available at: www.incb.org/documents/Psychotropics/conventions/convention_1971_en.pdf (accessess 30 September 2025).

[bibr123-02698811251382147] Volonnino G La Russa R Di Fazio N , et al. (2023) Z-drugs and their use in drug-facilitated crimes: A review of the literature. La Clinica Terapeutica 174(5): 451–468. 37750379 10.7417/CT.2023.2465

[bibr124-02698811251382147] Warren AL Lankri D Cunningham MJ , et al. (2024) Structural pharmacology and therapeutic potential of 5-methoxytryptamines. Nature 630(8015): 237–246. 38720072 10.1038/s41586-024-07403-2 PMC11152992

[bibr125-02698811251382147] Weil AT Davis W (1994) Bufo alvarius: A potent hallucinogen of animal origin. Journal of Ethnopharmacology 41(1–2): 1–8. 8170151 10.1016/0378-8741(94)90051-5

[bibr126-02698811251382147] Wilkes R Roberts DM Liknaitzky P , et al. (2024) The psychedelic call: Analysis of Australian Poisons Information Center calls associated with classic psychedelics. Clinical Toxicology (Philadelphia, PA) 62(4): 242–247. 38753585 10.1080/15563650.2024.2346612

[bibr127-02698811251382147] Willins DL Meltzer HY (1997) Direct injection of 5-HT2A receptor agonists into the medial prefrontal cortex produces a head-twitch response in rats. The Journal of Pharmacology and Experimental Therapeutics 282(2): 699–706. 9262333

[bibr128-02698811251382147] Winter JC Filipink RA Timineri D , et al. (2000) The paradox of 5-methoxy-N,N-dimethyltryptamine: An indoleamine hallucinogen that induces stimulus control via 5-HT1A receptors. Pharmacology, Biochemistry and Behavior 65(1): 75–82. 10638639 10.1016/s0091-3057(99)00178-1

[bibr129-02698811251382147] Winter JC Eckler JR Rabin RA (2004) Serotonergic/glutamatergic interactions: The effects of mGlu2/3 receptor ligands in rats trained with LSD and PCP as discriminative stimuli. Psychopharmacology 172(2): 233–240. 14598016 10.1007/s00213-003-1636-2

[bibr130-02698811251382147] Yanagita T (1986) Intravenous self-administration of (−)-cathinone and 2-amino-1-(2,5-dimethoxy-4-methyl)phenylpropane in rhesus monkeys. Drug and Alcohol Dependence 17(2–3): 135–141. 3743404 10.1016/0376-8716(86)90004-9

[bibr131-02698811251382147] Young R Rosecrans JA Glennon RA (1982) Comparative discriminative stimulus effects of 5-methoxy-N,N-dimethyltryptamine and LSD. Life Sciences 30(24): 2057–2062. 7109835 10.1016/0024-3205(82)90446-5

[bibr132-02698811251382147] Zeifman RJ Singhal N Breslow L , et al. (2021) On the relationship between classic psychedelics and suicidality: A systematic review. ACS Pharmacology & Translational Science 4(2): 436–451. 33860173 10.1021/acsptsci.1c00024 PMC8033757

[bibr133-02698811251382147] Zeifman RJ Yu D Singhal N , et al. (2022) Decreases in suicidality following psychedelic therapy: A meta-analysis of individual patient data across clinical trials. The Journal of Clinical Psychiatry 83(2): 21r14057. 10.4088/JCP.21r14057 35044730

